# Metabolic and transcriptomic analysis of Huntington’s disease model reveal changes in intracellular glucose levels and related genes

**DOI:** 10.1016/j.heliyon.2017.e00381

**Published:** 2017-08-30

**Authors:** Gepoliano Chaves, Rıfat Emrah Özel, Namrata V Rao, Hana Hadiprodjo, Yvonne Da Costa, Zachary Tokuno, Nader Pourmand

**Affiliations:** Biomolecular Engineering Department, University of California Santa Cruz, 1156 High Street, Santa Cruz, CA 95064, USA

**Keywords:** Medicine, Neurology, Biochemistry, Cell biology, Neuroscience

## Abstract

Huntington’s Disease (HD) is a neurodegenerative disorder caused by an expansion in a CAG-tri-nucleotide repeat that introduces a poly-glutamine stretch into the huntingtin protein (mHTT). Mutant huntingtin (mHTT) has been associated with several phenotypes including mood disorders and depression. Additionally, HD patients are known to be more susceptible to type II diabetes mellitus (T2DM), and HD mice model develops diabetes. However, the mechanism and pathways that link Huntington’s disease and diabetes have not been well established. Understanding the underlying mechanisms can reveal potential targets for drug development in HD. In this study, we investigated the transcriptome of mHTT cell populations alongside intracellular glucose measurements using a functionalized nanopipette. Several genes related to glucose uptake and glucose homeostasis are affected. We observed changes in intracellular glucose concentrations and identified altered transcript levels of certain genes including *Sorcs1, Hh-II and Vldlr*. Our data suggest that these can be used as markers for HD progression. *Sorcs1* may not only have a role in glucose metabolism and trafficking but also in glutamatergic pathways affecting trafficking of synaptic components.

## 1 Introduction

Huntington’s Disease (HD) is a progressive, autosomal dominant neurodegenerative disease that produces physical, mental and emotional changes due to loss of critically important brain neurons. The genetic basis for HD is an expansion of cytosine-adenine-guanine (CAG) repeats in the huntingtin (*HTT* or *IT15*) gene that leads to the formation of a prolonged polyglutamine (polyQ) tract in the N-terminal region of the mutant huntingtin protein (mHTT). The wild type huntingtin protein (HTT) is important for the intracellular transport and trafficking of proteins, organelles and vesicles. The expansion of glutamine (>36 repeats) produces a gain of function mHTT that affects the healthy function of cellular machinery, ultimately resulting in neurotoxicity and detrimental cell lethality in the brain [1].

Considerable research efforts have been made to elucidate the molecular and cellular mechanisms underlying HD pathology. The proposed mechanisms through which mHTT causes neurodegeneration include mutant protein aggregation, vesicle association, elevated oxidative stress, excitotoxicity, mitochondrial and transcriptional dysregulation [2, 3]. Evidence for several of these mechanisms has been found in post-symptomatic disease models or post-mortem brain samples. Controversially, during the pre-symptomatic stage of HD, cellular architecture and morphology have been found to be disrupted but neurodegeneration has been found to be minimal or absent [4, 5]. These studies have been limited to immunohistochemistry, fluorescence staining and immunoblot analysis, and provided static information of HD [6, 7]. However, little is known at the early stages of the disease about the dynamics of metabolic and transcriptomic changes, which could be instrumental not only for HD therapy for diagnosed patients but also for at-risk individuals. Striatum, a subcortical part of the forebrain, is the most vulnerable part of the brain in HD, almost disappearing during the course of disease. One of the major clinical symptoms of HD is the loss of the medium spiny neurons in the striatum [8]. Therefore, although genome-wide gene expression studies have been conducted on targeted affected tissues in the post mortem human brain, it has proven difficult to detect specific changes in the human striatum [9]. To expand our understanding of changes in the transcriptional landscape of the affected cells, mRNA expression profiling was performed on cells isolated from the striatum of rat embryos expressing wild type and mutant HTT.

As shown by Lin and Beal, detecting changes in carbohydrates can be instrumental for the development of useful therapies that slow down HD progression and reduce its severity at the early stages of the disease [10]. Recent evidence suggests that, similar to other neurodegenerative diseases, mice exhibit increased deregulation in brain energy metabolism at the late stages of HD [11]. A super-family of glucose transporter genes, the *GLUT* gene family, is responsible for the transport and uptake of glucose [12, 13]. The *GLUT* family includes 12 genes, each encoding one GLUT protein. Various members of this gene family were detected in the brain, although glucose transporter protein type I exclusively mediates transport across the blood-brain barrier [14, 15]. GLUT1, initially thought to be the only glucose transporter protein expressed in the brain, supports the basal metabolic needs of proliferating cells [16]. GLUT2 is primarily GLUT protein expressed in the pancreatic β-cells, liver and kidneys. In the pancreas, GLUT2 is believed to act in conjunction with glucokinase in the glucose-sensing mechanism. GLUT4 is the insulin-responsive receptor and is primarily expressed in the heart, adipose tissue, and skeletal muscle, where it is responsible for the reduction in the glucose levels in the post-prandial rise of glycaemia. Insulin stimulates the translocation of intracellular vesicles containing GLUT4 to the plasma membrane, resulting in a 10- to 20-fold increase in glucose transport [17].

Current assessment methods for intracellular glucose levels in HD models are limited and performed with a combination of techniques including gas chromatography-mass spectrometry (GC-MS), liquid chromatography-mass spectrometry (LC-MS), uptake assays, and electrophysiology [18]. The invasiveness, low sensitivity, and specificity of these techniques prevent longitudinal study of biological model cells. Other major limitations of these techniques are the complex sample preparation methods during which cellular concentration of glucose can be altered. Therefore, accurate real time detection of glucose starting from the early and going to the later stages of the disease may provide biochemical evidence that can inform the design of novel drugs and therapeutic regimens. We recently reported the use of nanopipette technology for intracellular measurements [19, 20]. Importantly, because nanosensors are minimally invasive and not destructive to the cell, it is possible to take repeated measurements of the same cell. In this work, we employed glucose nanosensors for intracellular glucose measurements in HD cell models (ST14A) at different time points to investigate glucose metabolism over time. In parallel, we conducted transcriptomic analysis to understand temporal changes in RNA expression in model HD cells. We report impaired glucose metabolism in single rat striatum mHTT cells using our nondestructive nano-glucose sensor. This is the first report determining the intracellular glucose levels in mHTT cells to be in the range of 0.8–1.5 mM. A 2.5 fold decrease in intracellular glucose levels in HD cells indicated an altered glucose metabolism that was further investigated by transcriptomic study. Significant differences were seen in glucose uptake and glucose homeostasis genes. Interestingly, there was an increased expression of *Sorcs1* in mHTT cells which has been previously associated with late onset of Alzheimer’s disease, another neurodegenerative disorder. Overexpression of *Sorcs1* was confirmed by performing RT-qPCR which corroborates *Sorcs1* can be used as a biomarker for HD progression.

## 2 Materials and methods

### 2.1 Reagents

Glucose Oxidase (GOx) from Aspergillus niger ≥100,000 units/g (Type VII, lyophilized, EC Number 232-601-0), ferrocene (98%), poly-l-lysine (PLL) (0.1% solution in water), glutaraldehyde (GA) (25% in water) and D-glucose were purchased from Sigma Aldrich (St. Louis, MO). Silver wires (125 μm) were supplied from A-M Systems (Sequim, WA). Glucose free Dulbecco’s Modified Eagle Medium (DMEM), trypsin (0.25%, phenol red) and penicillinstreptomycin were bought from Gibco while Hank’s Balanced Salt Solutions and fetal bovine serum were purchased from GE Healthcare (GE Healthcare). 500 μm gridded plates were obtained from Ibidi (Ibidi USA, Inc., Madison, WI).

### 2.2 Glucose nanosensor fabrication

Glucose nanosensors with an outside diameter of 1.00 mm and an inside diameter of 0.70 mm were fabricated from quartz capillaries (Sutter Instrument, Novato, CA) using a P-2000 laser puller (Sutter Instrument, Novato, CA) as described elsewhere [19]. Briefly, to create glucose nanosensors, the nanopipettes were modified as follows: Pulled nanopipettes were backfilled with a 15 μl solution containing 10 mM Ferrocene prepared in 100 mM PBS (pH 7, supplemented with 0.1 M KCl). An Ag/AgCl electrode was placed into the nanopipette as a working electrode while another Ag/AgCl electrode was immersed in the cell media as a reference/counter electrode. Nanopipettes were modified with PLL by backfilling the nanopipette interior. Then nanopipette surface was treated with a 10% (v/v) solution of glutaraldehyde (GA) for 30 min. GOx (900 mU) was then reacted with the activated nanopipette walls. All glucose nanosensors were calibrated in DMEM with standard glucose concentrations.

### 2.3 Cell culture

The Huntington’s disease model cell lines that were used in this work are both striatal cell lines derived from rat embryos and obtained from Coriell Institute cell repository. The two cell lines express human HTT fragments of 15 and 120 polyglutamine repeats representing wild type (ST14A-Q15) and mHTT disease model (ST14A-Q120), respectively. All cells were cultivated and maintained in DMEM (supplemented with 10% fetal bovine serum, antibiotics) with various glucose concentrations. DMEM was supplemented with 1 nM insulin when needed to study effects of insulin.

### 2.4 Intracellular glucose measurement

The intracellular glucose measurement setup consists of an inverted microscope Olympus IX 70 with Spot Insight CMOS camera to image cells. The nanosensors are fixed to a microscope by a pipette holder (Axon Instruments). The holder is connected to an Axopatch 700B amplifier (Molecular Devices) for current measurement, an MP-285 micromanipulator (Sutter Instrument) for coarse control of the nanopipette positioning in the *X, Y*, and *Z* directions, a Nanocube piezo actuator (Physik Instrument) for fine control in the *X*, *Y*, and *Z* directions, and a PCIe-7851R Field Programmable Gate Array (FPGA) (National Instruments) for hardware control of the system. The system is operated using custom-coded software written in LabVIEW. Current-clamp technique has been used at 1nA with signal filter at 1 kHz. The signal was further digitized by an Axon Instruments Digidata 1322A. The data were then recorded by a LabVIEW 9.0 home-made software, as described previously [21]. While in the medium, a fixed potential of 500 mV was applied to the glucose nanosensor. This biased potential generates an ion current through the liquid-liquid interface that is used as the input into a feedback loop analyzed by the scanning ionic conductance microscope. When a 5–10% current decrease is detected, the glucose sensor is penetrated into the target cell up to 0.8 μm at 100mm/s. The sensor is maintained inside the target cell for a pre-defined time of 60 seconds and then withdrawn to the initial position. Each condition was tested on a minimum of 9 cells. Three consecutive readings were recorded for each individual target cell to ensure reproducibility and robustness. In order to investigate underlying molecular mechanism of these observed changes, a detailed transcriptome study was performed.

### 2.5 RNA isolation from HD cells

RNA extraction and purification were performed using RNeasy Mini kit from Qiagen. Prior to RNA isolation, HD cells were collected with conventional trypsin treatment and counted using Bio-Rad automated cell counter. A minimum of 5 × 10^5^ cells were used for RNA isolation. Samples were stabilized by treatment with RNAlater (Ambion). After RNA isolation, nucleic acid quantity and quality were checked using NanoDrop (Thermo Fisher Scientific).

### 2.6 RNA-seq library preparation, qRT-PCR and sequencing

#### 2.6.1 cDNA synthesis

Extracted total RNA was converted to cDNA using the Smart-seq2 protocol and the cDNA was preamplified as described by a previoud study [22]. Briefly, the cDNA product was mixed with KAPA HiFi HotStart ReadyMix (2X, KAPA Biosystems), ISPCR primer (10uM) and nuclease-free water (Gibco) and PCR amplified as follows: 98 °C 3 min, then 12 cycles of (98 °C 15 s, 67°C 20 s, 72 °C 6 min), with a final extension at 72 °C for 5 min. The cDNA was cleaned up using a 1X ratio of AMPure XP beads (Beckman Coulter).

#### 2.6.2 Quantitative real-time PCR

Real time PCR experiments were performed using 500 ng of reverse transcribed RNA and analyzed with SYBR green qPCR master mix (kapa Biosystems) using an Mx3000P instrument (Stratagene). The relative quantification of *Sorcs1* expression levels was performed using a previously described method [23]. For these experiments *actb* was used as a reference gene. *Sorcs1* and *actb* specific primer sequences designed in previous studies were used (Table 1).

**Table 1:** Sequences of primers used in this study.

GENE	FORWARD	REVERSE	REFERENCE
*Sorcs1*	5'-AGCCAACAGAAATAAACCTTTCC-3’	5'-TAATGTGGTCTTCTTCTTGATGT −3'	[76]
*β-Actin*	5’-CACCCGCGAGTACAACCTTC-3’	5’-CCCATACCCACCATCACACC-3’	[77]

#### 2.6.3 Library preparation and sequencing

The cDNA was diluted to ∼300 pg/ul and then processed with the Nextera XT DNA library preparation kit according to the manufacturer’s protocol (Illumina). These libraries were purified and size-selected with an average library size of 300–600 bp as determined by Bioanalyzer 2100 high-sensitivity DNA assay (Agilent).

Functional library concentration was determined with the KAPA Biosystems library quantification kit. The libraries were denatured after quantification and loaded on Illumina HiSeq 2000 for sequencing.

#### 2.6.4 Quality control and mapping of sequencing reads

Sequencing adapter sequences were removed from reads using Cutadapt version 1.4.2 [24]. Quality of preprocessed reads was evaluated using FastQC (http://www.bioinformatics.babraham.ac.uk/projects/fastqc/). The preprocessed reads were mapped as paired-end reads using the STAR pipeline [25] with default parameters against the UCSC rn5 *Rattus norvegicus* genome, combined with the human huntingtin gene (http://hgdownload.soe.ucsc.edu/goldenPath/rn5/bigZips/).

#### 2.6.5 Gene expression analysis

HTSeq package was used to generate the gene matrix from the output sam file produced by the STAR alignment. A gtf file containing Ensembl identifiers as well as gene names was used for rat and human genes. For the ERCC genes, the gtf file from Thermo Fisher Scientific was downloaded from the company website (www.thermofisher.com). The gene matrix was then subjected to DESeq2 differential expression analysis, as described [26, 27, 28, 29]. Benjamini-Hochberg multiple testing adjustment was used as a method to deal with p-values that are low due to chance, and not because the measurement represents a significant value. False Discovery Rate values used were calculated by DESeq2 package. Hierarchical Clustering of differentially expressed genes was performed using the Pretty Heatmaps function in R (v. 3.2.3). Pearson coefficients were used as correlation coefficients for the Hierarchical Cluster Analysis heatmaps. The Panther classification system (www.pantherdb.org) was used to visualize the pathways in which differential genes were involved.

### 2.7 Statistical analysis

Analysis of Variance (ANOVA) and independent t-test were performed using Prism (GraphPad) software when indicated.

## 3 Results and discussion

The energy metabolism in presymptomatic and symptomatic HD individuals is known to be defective [30, 31]. Reduced glucose uptake in the striatum and cortex of HD patients prior to the onset of clinical symptoms has been previously revealed by positron emission tomography (PET) scanning. Additionally, this hypometabolism demonstrated a high correlation with the onset and progression of HD, independent of the extent of CAG expansion [32]. Recently, Besson et al. reported that the increase of glucose transporters was beneficial to HD pathology in a *Drosophila melanogaster* model [33]. However, due to the destructive nature of previously employed intracellular glucose measurement methods, a direct interrogation of temporal changes at the transcriptomic and metabolic level could not have been studied. In this work, we took a step forward by deploying functionalized nanopipettes as glucose sensors to measure changes in intracellular glucose concentrations over time, and performed RNA sequencing to evaluate concomitant changes in expression of genes related to glucose metabolism.

### 3.1 Glucose nanosensors reveal temporal intracellular glucose changes at HD cells

Direct monitoring of glucose in cells is difficult due to the small size of the biological specimen and the complexity of the intracellular environment. Glucose nanosensors are made of glucose oxidase (GOx) functionalized quartz nanopipette with ∼ 100 nm pores. Due to their small size, glucose nanosensors can be inserted into individual cells, providing a non-destructive and direct *in vitro* quantification tool. We have recently demonstrated the use of this nanosensor in conjunction with a customized cell finder at normal and cancer cells [19].

Typical physiological concentrations of glucose in biological systems are in the low-micromolar range [34]; intracellular glucose levels in HD cells is unknown. More importantly, to our knowledge, there are no reports on the assessment of temporal changes in intracellular glucose concentration in HD models, an assessment that could illuminate one aspect of the cellular pathophysiology of HD. To achieve the goal of measuring intracellular glucose over time, we placed nanosensors in the cytoplasm of wild type (WT) (ST14A-Q15) and HD (ST14A-Q120) cells.

Cells underwent consecutive passages in fresh culture medium and glucose measurements were made at the end (day 3 (T1), day 6 (T2), day 11 (T3), day 16 (T4)) of each passage. In the initial experimental conditions, WT and HD cells were cultured in low glucose media (LGM) and high glucose media (HGM) containing 0.5X and 1X (4.5 g/L) glucose respectively. Cells were also cultured in glucose-free media but neither WT nor HD cells survived in this condition. Therefore, measurements could not be conducted in the absence of glucose. It is important to note that the regular culture condition of these cells does not require or include insulin.

**Fig. 1:**
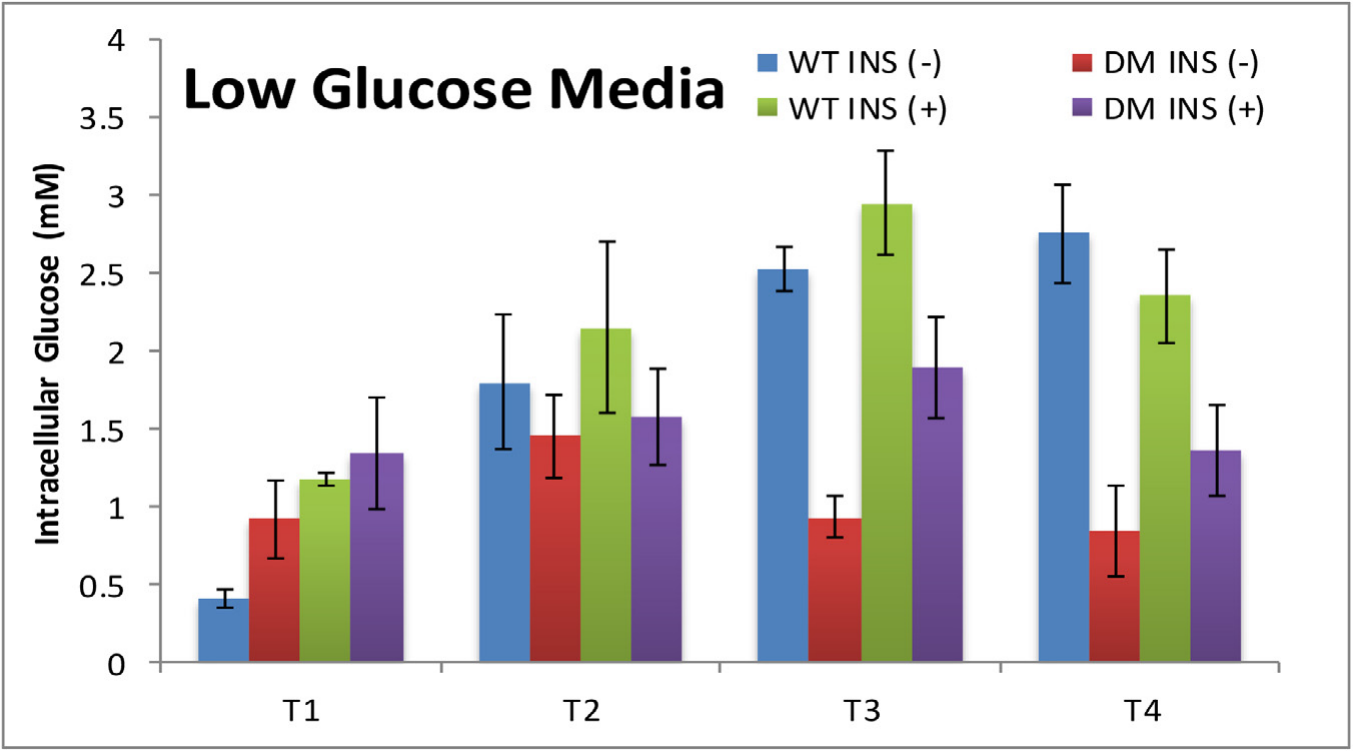
Bar graphs showing the average intracellular glucose concentrations measured using GOx-functionalized nanosensor in DMEM with low (LGM) glucose content in the absence of insulin (INS(−)) and 1 nM insulin (INS (+)) in wild type (WT) and disease cell lines (DM). Nine to 12 individual cells with 3 replicates were tested for each condition. The measurements were performed at day 3 (T1), day 6 (T2), day 11(T3), day 16 (T4).

**Fig. 2:**
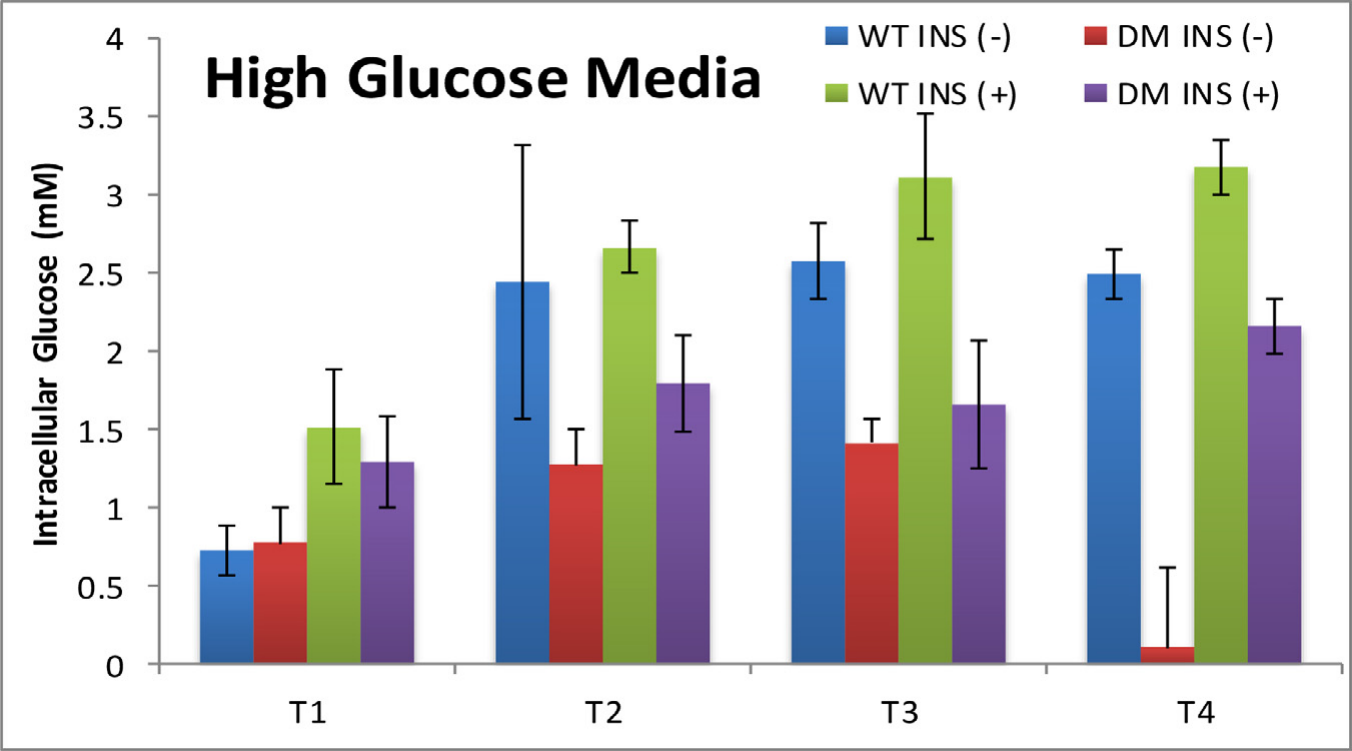
Bar graphs displaying the average intracellular glucose concentrations measured using GOx-functionalized nanosensor in DMEM with high glucose (HGM) content in the absence insulin (INS(−)) and 1 nM insulin (INS (+)) for wild type (WT) and disease cell lines (DM). Nine to 12 individual cells with 3 replicates were tested for each condition. The measurements were performed at day 3 (T1), day 6 (T2), day 11 (T3), day 16 (T4).

Exposure of WT cells to low and high glucose media in the absence of insulin resulted in similar intracellular glucose levels as measured by glucose nanosensors (Figs. 1 and 2 ). Intracellular glucose levels were found to be almost stable from T2 to T4, and the range was between 1.79 to and 2.58 mM. The most significant difference was observed for the T1 time point where intracellular levels were about 3 fold lower than those of T2-T4 in wild type cells. This low concentration can be due to either fast metabolism or rapid growth rate of young cells. When the glucose nanosensor was used on HD cells, we observed that intracellular levels of glucose were about 2.5 fold lower than that found WT cells for T3 and T4 (Figs. 1 and 2).

To compare glucose uptake in WT and HD cells, we exposed the cells to 1 nM insulin (normal non-fasting insulin level), as insulin regulates glucose uptake by activating glucose transporters 1 and 4 (GLUT1 and GLUT4) [35]. We saw a general increase in intracellular glucose levels for all time points for both cell lines (Figs. 1 and 2). As expected, in HGM, intracellular glucose levels were much higher than in LGM. Notably, the effect of insulin was more pronounced at T3 and T4 suggesting that glucose uptake of HD cells is facilitated when insulin is present. The average intracellular glucose concentrations and their variations for both cell types under all conditions are summarized in Table 2.

**Table 2:** Summary of intracellular glucose measurements at wild type (WT) and disease (HD) cells in the absence (INS −) and presence (INS +) of insulin. The standard deviations (σ) were calculated for *n = 9* replicate measurements for each condition (σ = (1/N(Σ(*χ_i_− μ)^2^)^1/2^*).

		WT	HD	WT	HD	WT	HD	WT	HD
		T1		T2		T3		T4	
[Glucose] mM	LGM INS (-)	0.41 (±0.06)	0.92 (±0.25)	1.79 (±0.43)	1.45 (±0.26)	2.53 (±0.13)	0.93 (±0.13)	2.54 (±0.29)	0.84 (±0.29)
	LGM INS (+)	1.17 (±0.05)	1.34 (±0.35)	2.15 (±0.55)	1.58 (±0.31)	2.61 (±0.16)	1.89 (±0.33)	2.35 (±0.29)	1.36 (±0.29)
	HGM INS (-)	0.72 (±0.16)	0.77 (±0.23)	2.44 (±0.87)	1.28 (±0.23)	2.58 (±0.23)	1.42 (±0.16)	2.49 (±0.23)	0.11 (±0.51)
	HGM INS (+)	1.52 (±0.36)	1.29 (±0.29)	2.67 (±0.16)	1.80 (±0.31)	3.12 (±0.40)	1.66 (±0.40)	3.19 (±0.18)	2.16 (±0.17)

### 3.2 Assessing transcriptomics in rat HD cells

We integrated transcriptomics and metabolomics i.e., the metabolome provided phenotypic (glucose) measurements to which we anchored the global measurements of the transcriptome related to glucose pathways. In order to achieve this, RNA-seq transcriptomics was performed to complement the data obtained from glucose nanosensor measurements, using a similar set-up. RNA was extracted from wild type (WT) (ST14A-Q15) and disease (HD) (ST14A-Q120) cells. The gene expression levels were assessed across four passages T1-T4.

**Fig. 3:**
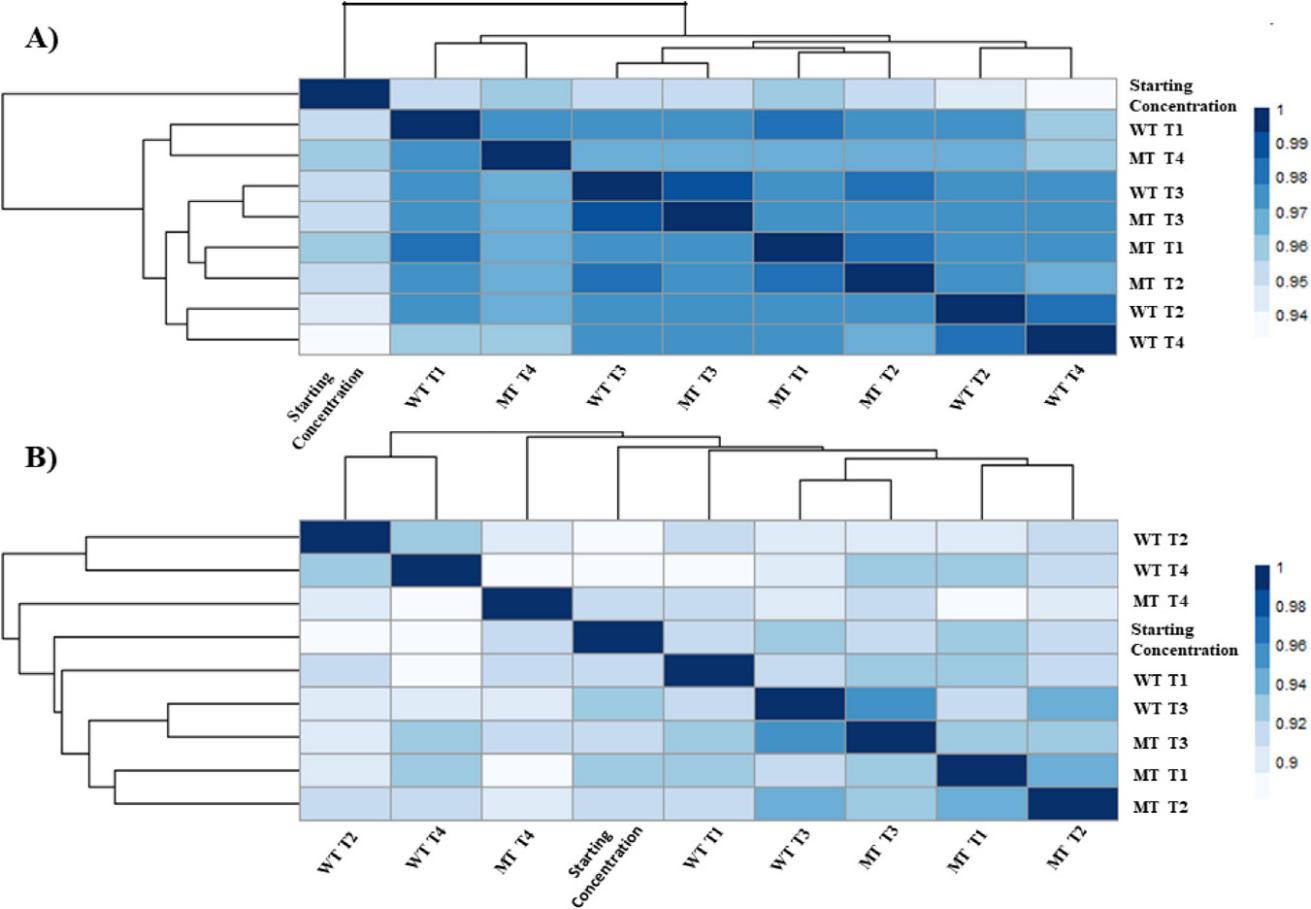
A) Pearson correlation of the starting ERCC spike-in concentration and the ERCC spike-in count after library preparation and RNA-seq. ERCC expression values were calculated as log2 of counts. B) Spearman correlation of the starting ERCC spike-in concentration and the ERCC spike-in counts after RNA-seq. ERCC expression values were calculated as log2 of counts.

First, to assess the sequencing by synthesis (SBS) method as a quantitative profiling tool, ERCC spike-in controls were included in the cDNA mix with the samples. ERCC spike-in controls showed Pearson correlation above 90% in all library-to-library comparisons. Also, a 90% Pearson correlation was observed in the expression of ERCC pseudo-genes to the ERCC starting concentrations provided by the ERCC manufacturer (Fig. 3 (A)). Although Spearman correlation was slightly lower (minimum 88%) (Fig. 3 (B)), Pearson correlation is a better parameter for our dataset because it assumes a linear relationship between ERCC gene expression measurements and ERCC starting concentration [36].

Comparisons of upregulated, downregulated and significantly different genes were made with the top 50 genes in a universe of 3213 differentially expressed genes. A recent RNAseq study in human post-mortem HD patients also showed 5480 genes differentially expressed, indicating a similar order of magnitude in the number of differentially expressed genes in rat and humans due to mHTT [9].

### 3.3 Glucose-related genes were differentially expressed in mHTT cells

**Fig. 4:**
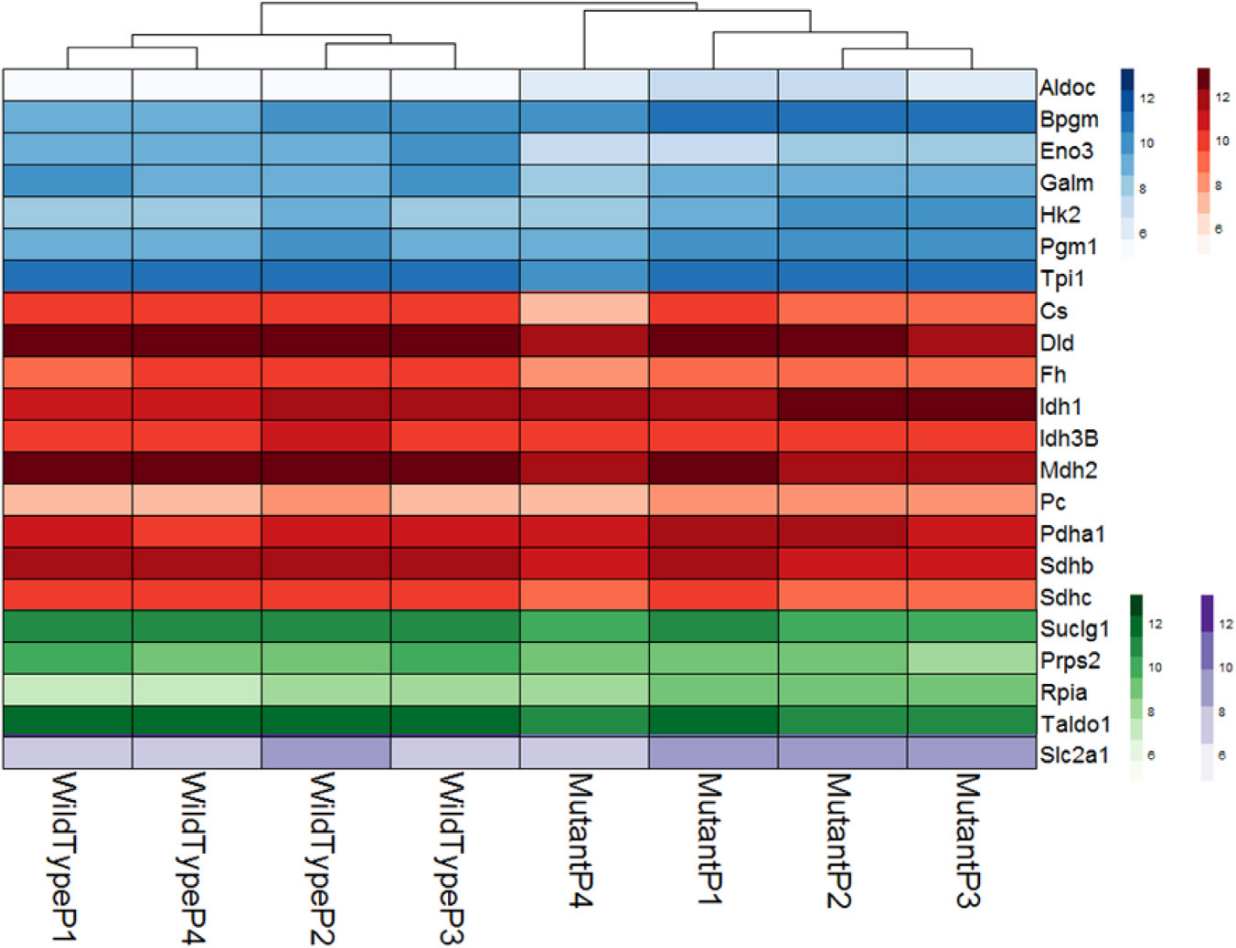
Glucose metabolism related genes derived from glycolysis (blue), PPP (red), TCA (green) and glucose transport pathways (purple) differentially expressed in wild type and HD cells. Statistical significance was determined by the Benjamini-Hochberg multiple testing adjustment, as described in DESeq2 documentation, based on p-value comparison. FDR was applied on p-values for significance cut-off.

Specific genes used for the analysis were involved in glycolysis, trichloroacetic acid cycle (TCA), pentose phosphate pathway (PPP) and glucose transport; accessed from a list of genes in glucose metabolism provided in Qiagen’s website (www.qiagen.com). The pattern of expression of genes involved in these pathways in mutant and wildtype were plotted as a heatmap using the Pheatmap package available in R [75]. Seven genes in the glycolysis pathway, eleven genes in the TCA, two genes in the PPP, and one gene expressing a glucose transporter were found to be affected by mHTT in this study (Fig. 4).

### 3.4 Survival effect of HK-II and Glut1 on HD cells

Hexokinase catalyzes the first step of glycolysis and represents a regulatory enzyme [74]. Expression of hexokinase II (HK-II) was found to be upregulated in our rat striatal HD cells, a finding also observed by other authors [37]. An increased expression of hexokinase II has also been associated with other neurodegenerative disorders [38] (Fig. 4). It was demonstrated previously that alteration in HK-II gene expression has a direct impact on glucose metabolism [39].

Hexokinases (HKs) phosphorylate the glucose transported through glucose transporters (GLUTs) on the plasma membrane to produce glucose-6-phosphate (G-6P) (Berg et al., 2002). HK activity is inhibited by G-6P providing a feedback mechanism [40]. The reduced glucose content measured in mutant cells could be attributed to the increased expression of HK-II in HD cells. HK-II upregulation has been previously shown to be an important consequence of metabolic re-programming in cancer [39].

The expression of *Glut1* (Slc2a1), the main class of glucose receptor involved in glucose transport from the blood-brain barrier to the CNS [15], was also upregulated in the mutant cell type (Fig. 4). A previous study in a hematopoietic cell line showed that increased glucose phosphorylation due to co-expression of HK-II and *Glut1* has an anti-apoptotic effect. The protective effect was attributed to increase in NADPH activity through the pentose phosphate pathway eliciting an anti-apoptotic effect [41]. The mutant huntingtin protein causes neuronal dysfunction and eventual cell death. Some of the pathways found to be abnormal in Huntington’s disease models are transcriptional impairment, neuronal excitotoxicity, oxidative damage, inflammation, apoptosis, and mitochondrial dysfunction. The anti-apoptic effect could slow down the disease progression in our HD rat cell model, similar to observations by the study of Johri and Beal [42]. In summary, our data suggest a compensation mechanism by which increased expression of *HK-II* and *Glut1*, may drive mutant cells to survive huntingtin mutation.

Insulin treatment is known to increase *HK-II* mRNA and protein in various cell types and the increase is blocked by inhibition of PI3K, an upstream kinase of Akt, as well as inhibition of mTORC1 [39]. mTORC1 is a protein that integrates signals to regulate cell growth and metabolism.

Genes coding for IDH1, ENO3, MDH2 and ALDOC proteins were also found to be differentially expressed (Fig. 4) in mHTT cells. *Eno3* is a gene that codes the enolase version of the muscle. The deficiency of this protein correlates with glycogen storage disease XIII [43]. Together, the results of this glucose homeostasis targeted analysis suggest that glucose metabolism is altered in our HD model, in agreement with other studies [44].

### 3.5 Upregulated and downregulated genes in HD rat cell model

**Fig. 5:**
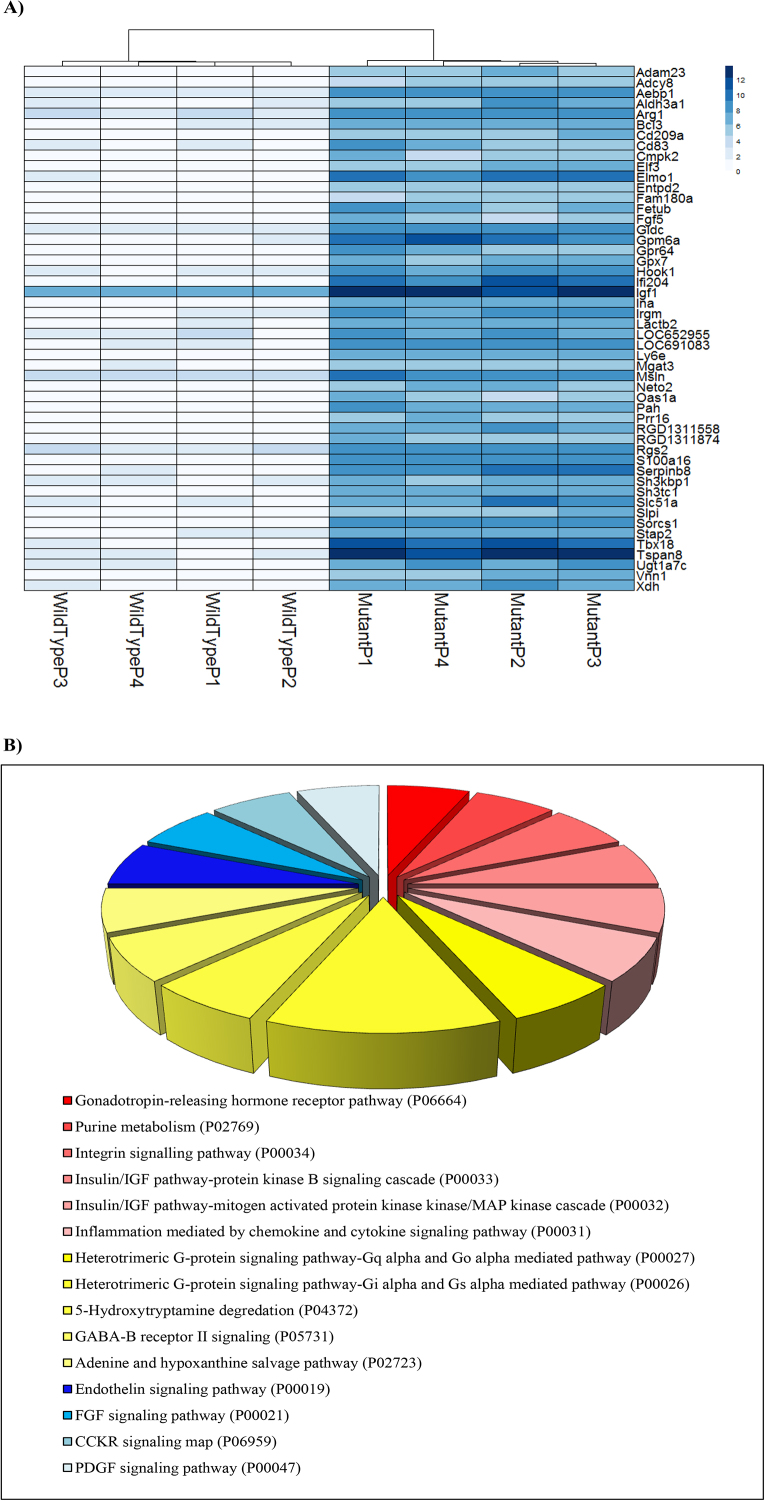
a) Top 50 up-regulated genes in the comparison log2 (value in mutant)/(value in wild-type), as described in the DESeq2 documentation. b) Pie chart showing Panther pathway analysis of the top 50 up-regulated genes of rat cells expressing human huntingtin.

To further understand the perturbation initiated by huntingtin, we analyzed the most abundantly differentiated genes of our cell model in the Panther Classification System (www.pantherdb.org). The group of the top 50 upregulated genes was segmented after the pathway enrichment analysis of genes present using Panther Classification. Fig. 5 shows the heatmap and pie-chart analysis of the up-regulated genes. Fig. 5b shows the main cellular pathways identified by the Panther Classification System among the top 50 expressed genes. They were identified to be cellular receptors controlling signal transduction, including EGF receptor, Wnt signaling, and glutamate receptor. Pathways related to Huntington’s and Alzheimer’s diseases were also found in the Panther Classification. Igf1 is involved in two pathways: the MAPK pathway, related to cell development and differentiation, and the AKT/PKB pathway that controls insulin metabolic actions (Fig. 5) [45].

SORCS1 protein is necessary for insulin granule secretion in β-cells [46], however was not found annotated with such function by the Panther Classification System in our analysis. SORCS1 is sortilin-related vacuolar protein sorting 10 (VPS-10) domain containing receptor 1. Interestingly, the gene expressing SORCS1 controls protein trafficking and transport of other cellular components, such as the amyloid beta plaques [47, 48], and has been established as an important hallmark of Alzheimer’s disease. Gene *Sorcs1* was found upregulated in the mutant type in this study (Fig. 5a). A recent study suggested that SORCS1 has a novel regulatory mechanism and acts as a modulator of sortillin function [49]. Sortillin, also a member of the Vps 10 protein sorting receptor family, is involved in biological processes such as glucose and lipid metabolism. It has been proposed that dysfunction of SORCS1 protein may contribute to both the APP/Aβ disturbance underlying Alzheimer’s disease (AD) and the insulin/glucose disturbance in Diabetes Mellitus (DM) [50].

**Fig. 6:**
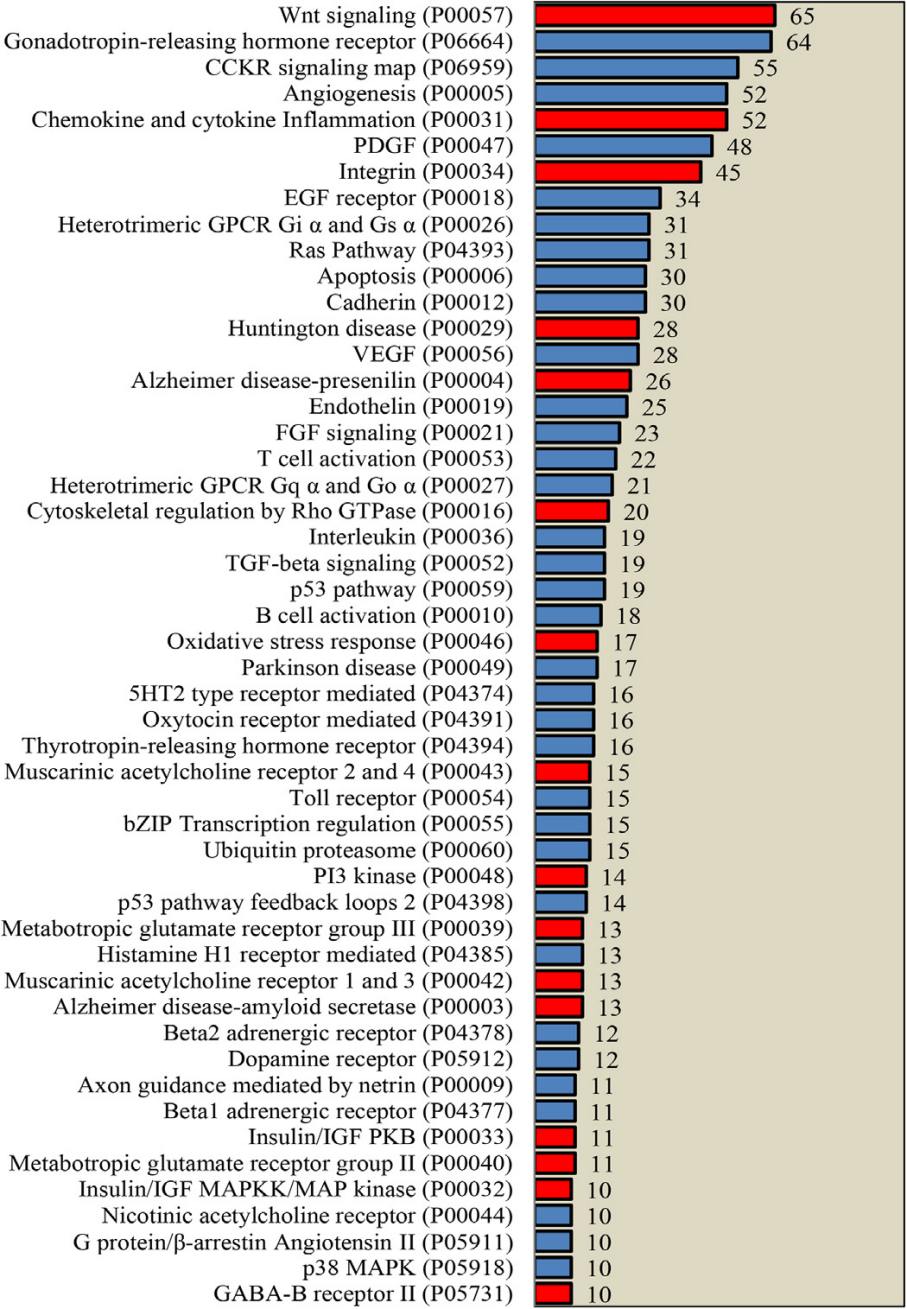
Number of differentially expressed genes per signaling pathway according to Pantherdb analysis. A minimum of 10 genes per signaling pathway was requested. Pathways were organized from highest to lowest number of differentially expressed genes within each pathway. Some of the pathways in red bars represent signaling pathways which are discussed in this manuscript.

**Fig. 7:**
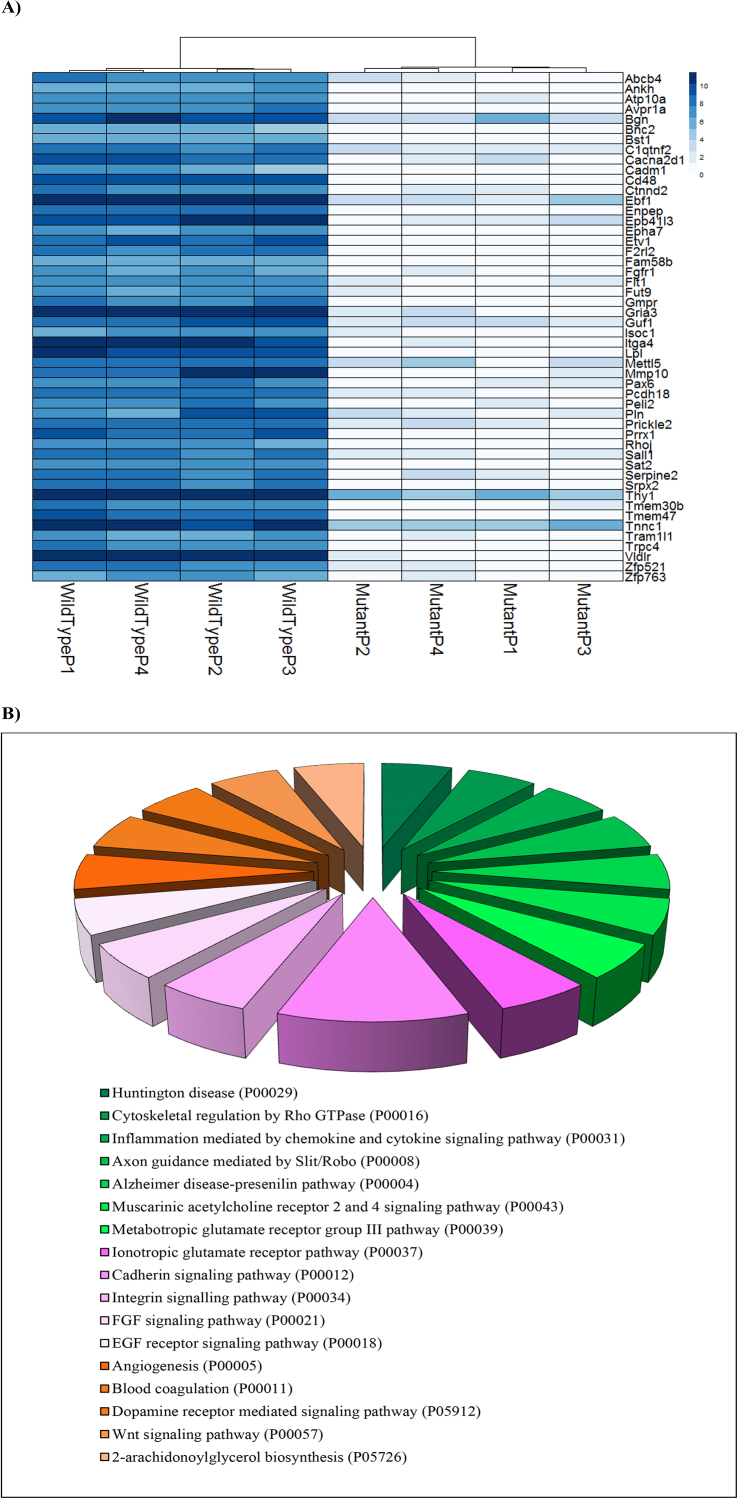
a) Top 50 down-regulated genes in the comparison log2 (value in mutant)/(value in wild-type), as described in the DESeq2 documentation. b) Pie chart showing Panther pathway analysis of the top 50 down-regulated genes of rat cells expressing human huntingtin.

To better investigate changes observed in mHTT model in this study, we compared transcriptomics data from other Huntington mice models and included some of the changes observed in AD. Out of 70 genes annotated in the Alzheimer’s disease presinilin pathway, 26 genes were found altered in mHTT cells (Fig. 5 and Fig. 6). The presinilin pathway is dysregulated in AD, cleaving several single-transmembrane proteins within the membrane domain, including the amyloid precursor protein, Notch, ErbB4, E-cadherin, Nectin-1alpha, and CD44 (www.pantherdb.org). Notably, another important AD marker, gene *Apoe*, was affected in our cell model. Interestingly, *Vldlr*, an *Apoe* receptor was the top downregulated gene in our study (Fig. 7). As mentioned before, HK-II, a glycolysis enzyme, was affected by the mHTT protein. Besides the already established low-glucose metabolism characteristic of AD, it was observed that AD condition affects the regulation of 6-phosphofructo-2-kinase in human patients, further illustrating common features between AD and HD [51].

**Fig. 8:**
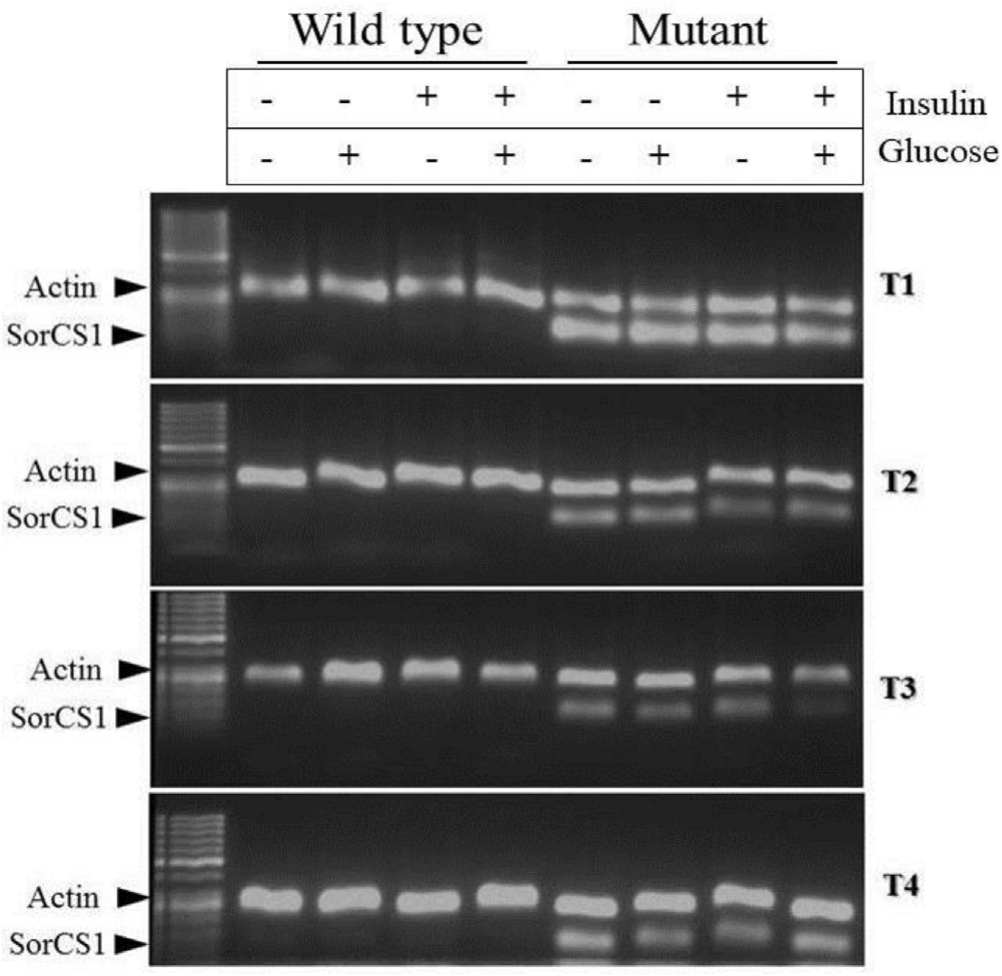
Polymerase Chain Reaction products of SorCS1 and actin primers amplification. 5μL of actin amplicons and 5 μL of SorCS1 amplicon were mixed and allowed to resolve in agarose gel electrophoresis as indicated above. Insulin and glucose treatments were indicated above each lane. Results suggest a higher expression of SorCS1 in the mHTT cell type, confirming RNA-seq data. The full, uncropped gel is available as Supplementary Fig. 6.

**Fig. 9:**
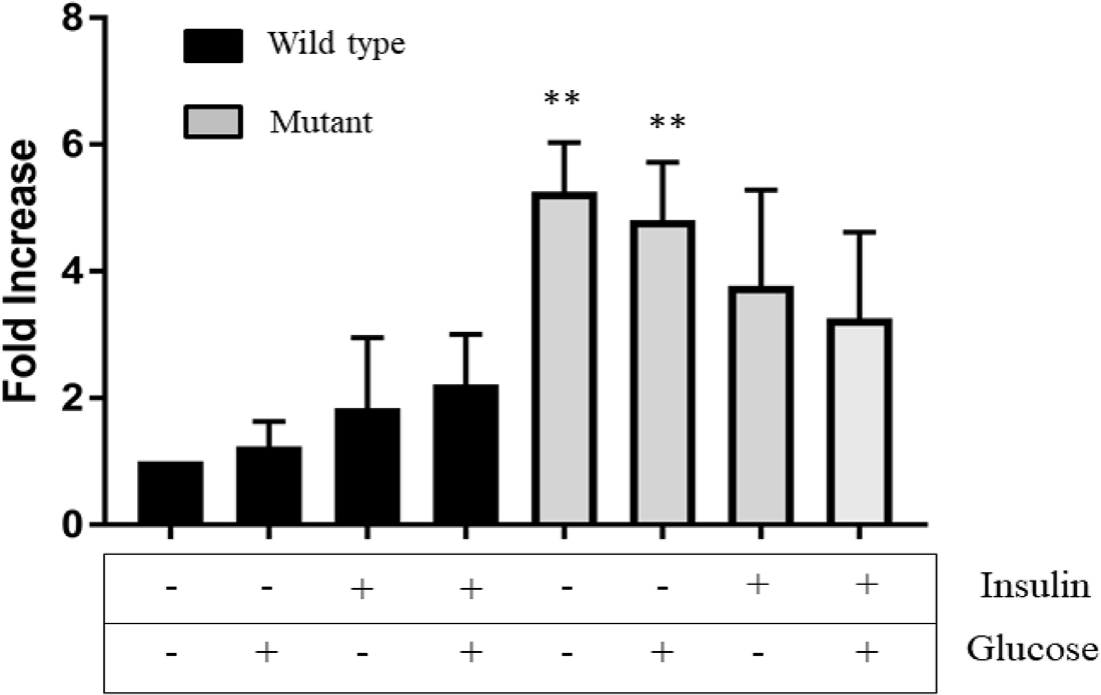
Relative gene expression (fold change) of *Sorcs1* gene in mHTT and wild type was normalized to reference condition (wild type cells grown in media containing no additional glucose or insulin). *actB* was used as endogenous control. mHTT cells had 2- 4-fold increase in *Sorcs1* levels. Results are presented as mean (T1, T2, T3 and T4) ± SE. Statistical analysis was assessed by unpaired t-test and one-way ANOVA. **P < 0.05 was considered statistically significant.

Other pathways significantly affected in our mHTT model, including calcium signaling, glutamate receptor activity and synaptic transmission, were observed by other researchers in HD mice and human patients [52, 53, 54, 55]. For instance, Achour and collaborators identified Gata2 transcription factor, a gene involved in establishment of tissue-specific enhancers, which was found to be affected in the present study, highly expressed in the striatum, and not cerebellum, of HD mice model [56]. Furthermore, genetic variations in intron 1 of *Sorcs1* have been associated with Alzheimer’s disease in several studies [57, 58]. In order to confirm upregulation of *Sorcs1* observed in RNA seq analysis, we stimulated cells with insulin as described in intracellular glucose measurements and performed gene specific PCR analysis (Fig. 8). *Sorcs1* was found to be upregulated in mHTT compared to wild type as observed in RNA-seq data. To understand the effect of insulin and/or glucose on expression of *Sorcs1* in mHTT cells, qRT-PCR was performed. The mHTT cells not treated with glucose and insulin showed the highest *Sorcs1* expression (Fig. 9). Significant difference was observed when these mHTT cells (no insulin, no glucose) were compared to WT cells grown in all conditions tested. However, we observed no statistically significant difference in *Sorcs1* expression in mutant cells treated with either insulin or glucose. Our results suggest that mHTT mutation was the only factor causing higher *Sorcs1* expression. However, more stringent and detailed analysis will enable better understanding of the effect of additional glucose and insulin on expression of *Sorcs1.*

In response to insulin, sortillins can transport GLUT4 to the membrane for uptake of glucose [59]. There are no previous studies demonstrating that the two proteins, SORCS1 and sortilin, can act identically. However, recent literature has indicated that the SORCS are sortilin-related CNS expressed proteins, and, as they belong to the same subgroup of VSP10 receptor family, these proteins are related [46, 47, 60]. Since SORCS1 and sortilins belong to the same family of proteins, we were interested in understanding whether increased *Sorcs1* in cells with Huntington phenotype would affect *Glut4* gene expression, thereby increasing glucose uptake.

**Fig. 10:**
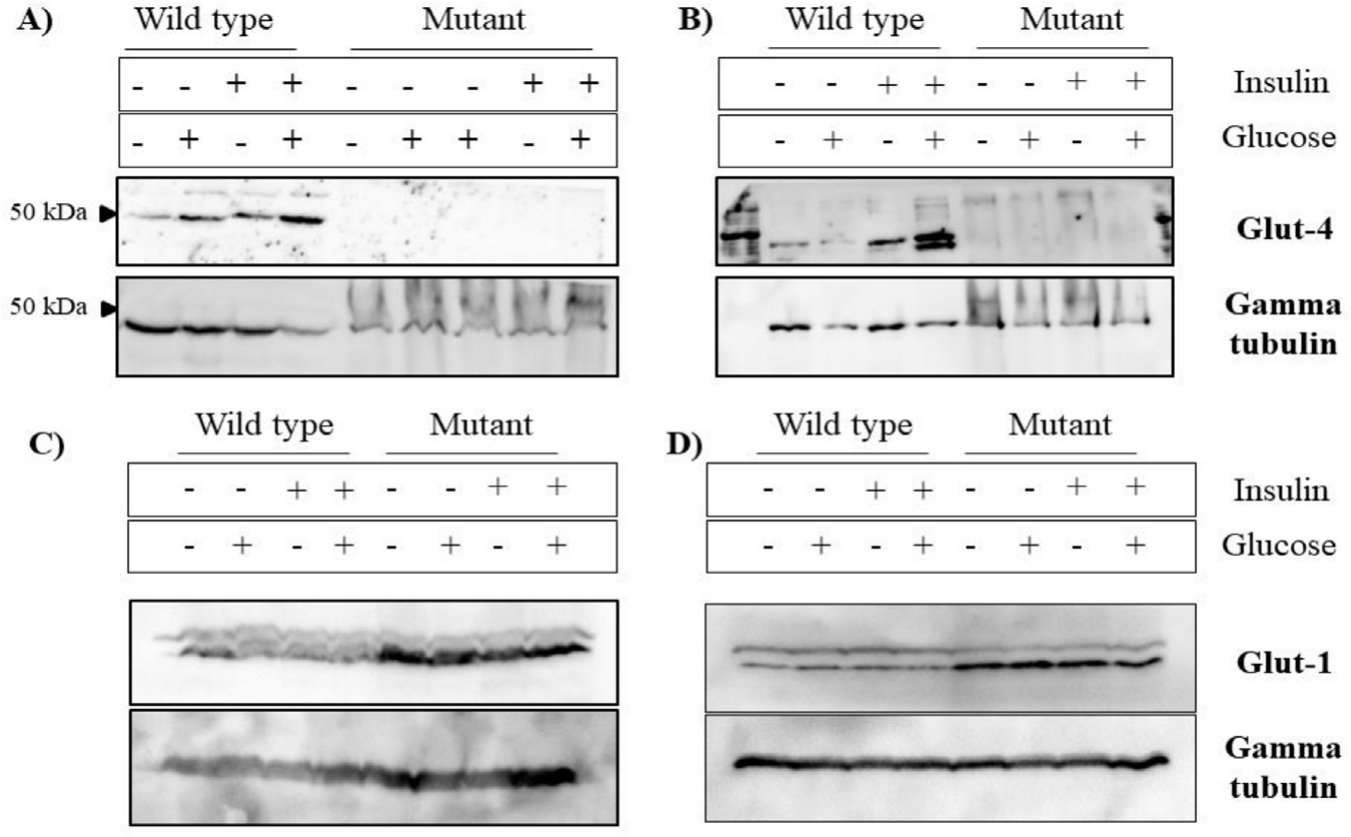
Western blot of glucose receptors. Glucose transporter 4 protein expression comparing mHTT and wild type HTT-expressing cell lines corresponding to passages 1 (T1) (A) and 4 (T4), (B) of ST14A cells. A decreased GLUT4 expression is observed due to mHTT. C) Glucose transporter 1 protein expression comparing mHTT and wild type HTT-expressing cell lines corresponding to passages 1 (T1) (C) and 2 (T2), (D) of ST14A cells. Mutants (mHTT cells) had an increased expression of GLUT1 receptor. The full, uncropped blots are available as Supplementary Figs. 8A1, 8A2, 8B1, 8B2, 8C1, 8C2, 8D1, 8D2.

As *Glut4* expression was not significantly different in HD and normal cells in the RNA-Seq analysis, we performed protein expression studies. Our results showed that GLUT4 protein was expressed at lower level in rat HD cells (Fig. 10A and B)**.** Basal levels of GLUT4 along with other glucose receptors including GLUT1, the main class of glucose receptor, may compensate for the lack of GLUT4 protein observed in mHTT cells. Nevertheless, other factors including post-transcriptional modification or action of miRNA may also have an effect on decreased GLUT4 protein levels, which needs to be investigated further. Hou and Pessin reported that overexpression of sortillin stabilizes GLUT4 protein, increases the formation of insulin responsive compartments, and promotes insulin-stimulated glucose uptake [59]. Higher GLUT4 and/or higher metabolism could explain the increased intracellular glucose concentration in wild-type cells and a time-dependent study could give a better idea whether GLUT4 is indeed regulated by SORCS1 in addition to sortillin, as observed in the study by Hou and Pessin [59]. As GLUT4 levels were lower in mHTT cells, we became interested in analyzing GLUT1 levels, the main class of glucose receptor. Results showed higher GLUT1 levels in mHTT cells suggesting that GLUT1 could indeed compensate for the lack of Glut4 in mHTT cells (Fig. 10C)**.**

The fine regulation of brain developed neutrophic factor (BDNF) by sortillin has been implicated in neuronal and tumor cell survival. Interestingly, BDNF protein was also shown as a diabetes and Huntington’s disease marker [2, 61]. In our cell model, following the trends described by Zuccato and collaborators, *Bdnf* levels were lower in mutant cells than in wild type. Furthermore, it has been demonstrated that BDNF protein is one of the molecules that is a target of organelle sorting mediated by SORCS1 [62]. BDNF, once transported to the striatum from the cortex, induces cholesterol synthesis and is related to synaptic plasticity and neuronal survival [63]. Sortillin has also been implicated in LDL-cholesterol metabolism and VLDL secretion [64, 65]. The very low density lipoprotein receptor (*Vldlr*) was found as the top downregulated gene in HD cells as compared to wild type cells in our data set (Fig. 7). VLDLR is an apolipoprotein E (APOE) receptor and the interaction of these two proteins has important implications in lipid metabolism [66]. Downregulation of *Vldlr* gene indicates that lipid metabolism is also impaired in mutant huntingtin striatal cells, as has been shown by another group [67]. Other genes that were found to be downregulated in this study are shown in Fig. 7a, along with the pathways they belonged to in Fig. 7b.

Together, these data suggest the involvement of *Sorcs1*, insulin, *Glut4* and *Glut1* in the HD condition, at least in our cell model, providing molecular candidates that support a decreased intracellular glucose concentration due to mutant huntingtin. To our knowledge, no other group has reported the involvement of *Sorcs1*, an Alzheimer’s disease and diabetes marker in a Huntington’s disease model. *Sorcs1* may not only be involved in impairment of glucose metabolism but also lipid metabolism in Huntington’s disease.

### 3.6 Other pathways affected by mutant huntingtin

Using transcriptomics and metagenomics, we sought to understand how mHTT influences not only HD related pathways but also other major pathways. In order to understand the pathways affected, we classified differentially expressed genes according to their signaling pathways, with at least 10 genes observed per pathway in the Pantherdb classification system. 1145 genes (out of 3213) are represented in Fig. 6. The signaling pathway most significantly affected by mutated huntingtin in number of genes was the wingless-related (Wnt) signaling pathway (P00057), showing 65 disregulated genes. Beta-catenins, a canonical element from the Wnt pathway, forms a complex with GSK-3B; they regulate epidermal growth factor and insulin, as well as control cell fate. Other genes that were differentially expressed were important in the insulin/MAPK/PKB/IGF pathways (P00032 and P00033). 64 genes were impaired in the gonadotrophin-releasing hormone receptor (P06664). Dysregulation of inflammation mediated by the cytokines and chemokines pathway (P00031) supports the anti-inflammatory treatments suggested for human HD [9]. Glutamatergic pathways were also found to be affected in our study (P00037 and P00039). Glutamate ionotropic receptor AMPA type subunit 3 (gria 3), a gene in this pathway, was downregulated in our study (Fig. 7)**.** Morton et al. also showed this gene to be downregulated in R2/6 mice, affecting synaptic plasticity [68]. A recent study showed SORCS1 regulating the AMPA receptors [47]. All the other pathways affected due to mutant huntingtin (containing 10 or more than 10 genes per pathway) are shown in Fig. 6. In summary, data shown on Fig. 6 support general observations from the literature about Huntington’s disease, as well as provide support to our observation of *Sorcs1* being dysregulated due to huntingtin mutation, in that this gene interacts with glutamate receptors [47].

## 4 Conclusion

Correlation between HD and glucose blood levels has been extensively studied. Although some studies do not show a correlation between HD and glucose metabolism, [3], other studies suggest the involvement of insulin resistance mediated by the huntingtin mutation and cross-talk between glucose metabolism and other metabolic pathways. Among these pathways, involvement of fatty-acid biosynthesis and cholesterol synthesis are described in HD progression [63, 67, 69, 70, 71, 72, 73]. Here, we showed impaired glucose metabolism in rat striatum cells using a functionalized nanopipette as a non-destructive intra-cellular glucose measurement technique. Impaired glucose metabolism along with altered expression of genes concerned with energy metabolism contributes to HD pathogenesis. A new additional target, *SorCS1* was identified to be altered in HD cells along with previously reported transporter related genes, glut1 and glut4. Increased levels of SorCS1 correlate positively with loss of the ability of the cell to sort proteins like glut4 properly. Further work on generating a knock-out mutant of *SorCS1* will shed more light on better understanding the role of this gene in Huntington’s disease. Glut1 and HK-II expression may have a beneficial effect on HD pathology by delaying apoptosis. The induction of genes in the PPP could possibly delay HD progression, thereby providing efficient neuroprotection against reactive oxygen species. These data represent a comprehensive integration of metabolic and transcriptomic expression data in rat HD cells. This research yields important advances in our understanding of HD pathogenesis. It remains to be shown whether the changes observed here are similar across different cell types within the brain of affected human individuals.

## Declarations

### Author contribution statement

Gepoliano Chaves, Rifat Emrah Ozel: Conceived and designed the experiments; Performed the experiments; Analyzed and interpreted the data; Wrote the paper.

Namrata Rao V: Performed the experiments; Analyzed and interpreted the data; Wrote the paper.

Hana Hadiprodjo: Analyzed and interpreted the data.

Yvonne Da Costa, Zachary Tokuno: Performed the experiments.

Nader Pourmand: Conceived and designed the experiments; Analyzed and interpreted the data; Contributed reagents, materials, analysis tools or data; Wrote the paper.

### Funding statement

This work was supported in part by grants from the National Institutes of Health [P01-35HG000205], National Institute of Neurological Disorders and Stroke [R21NS082927], Prize money awarded through the NIH Follow That Cell Challenge, internal support from UCSC Jack Baskin Engineering Dean and CAPES, Coordination for the Improvement of Higher Education Personnel - Brazil.

### Competing interest statement

The authors declare no conflict of interest.

### Additional information

Supplementary content related to this article has been published online at http://dx.doi.org/10.1016/j.heliyon.2017.e00381.

No additional information is available for this paper.

## Appendix A Supplementary data

Supplementary Figure6

Supplementary Figure 8A1

Supplementary Figure 8A2

Supplementary Figure 8B1

Supplementary Figure 8B2

Supplementary Figure 8C1

Supplementary Figure 8C2

Supplementary Figure 8D1

Supplementary Figure 8D2
