# Supplementary figures and images for: Metabolic and transcriptomic analysis of Huntington’s disease model reveal changes in intracellular glucose levels and related genes
Source: Heliyon. 2017 Aug 30;3(8):e00381. doi: 10.1016/j.heliyon.2017.e00381 (PMC5576993; doi:10.1016/j.heliyon.2017.e00381)

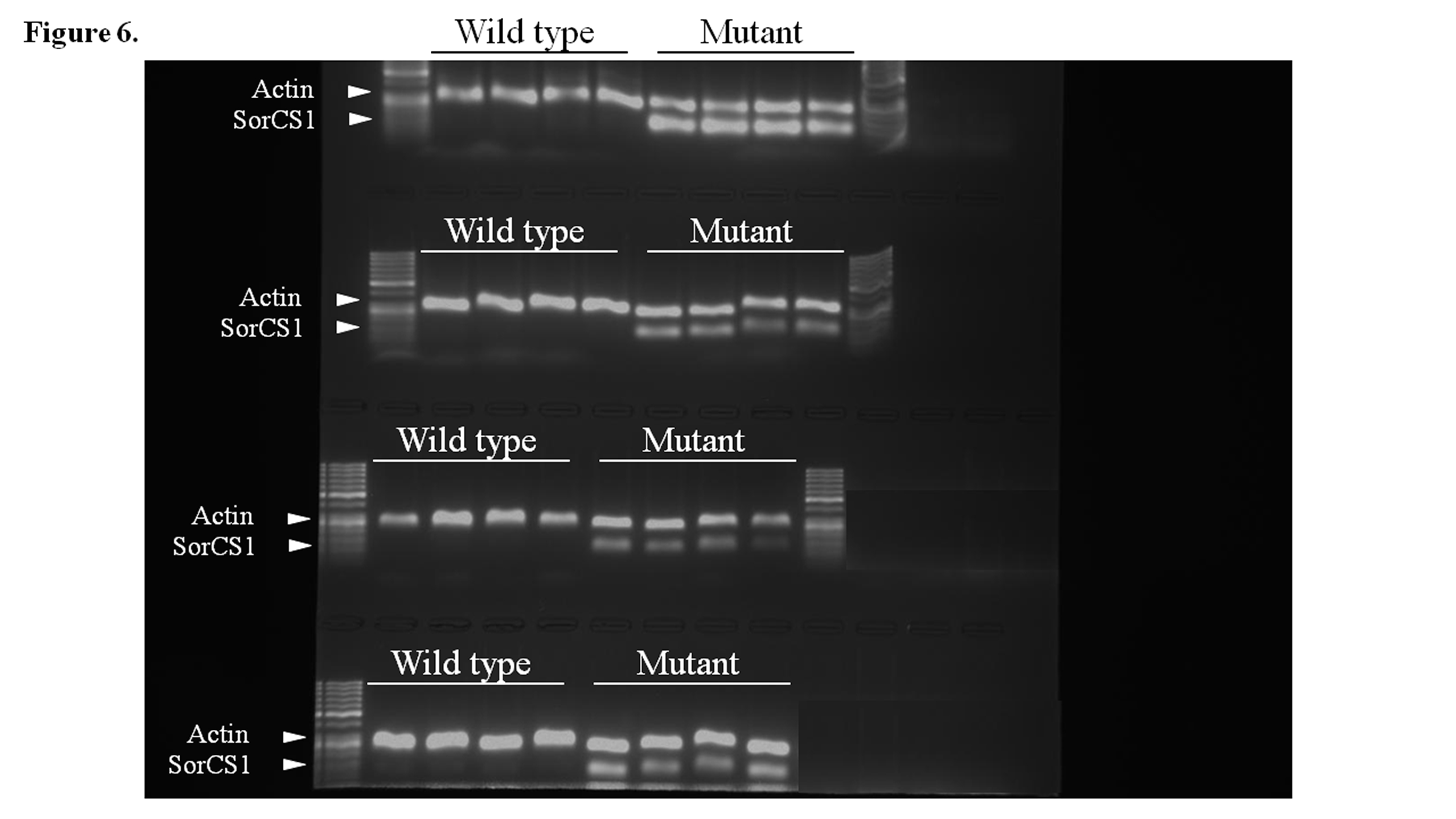

Supplement: Supplementary Figure6 [file mmc1.jpg]

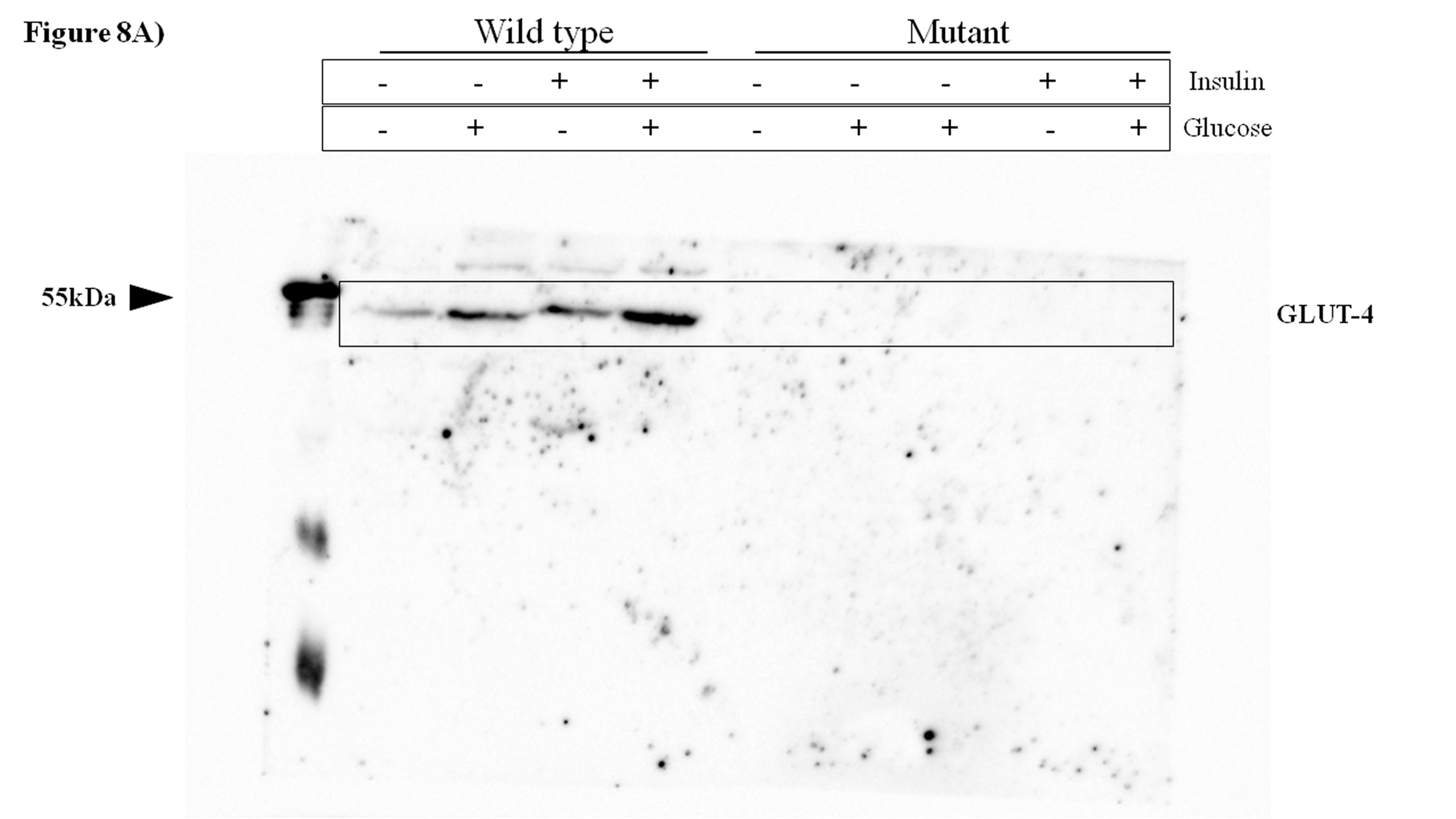

Supplement: Supplementary Figure 8A1 [file mmc2.jpg]

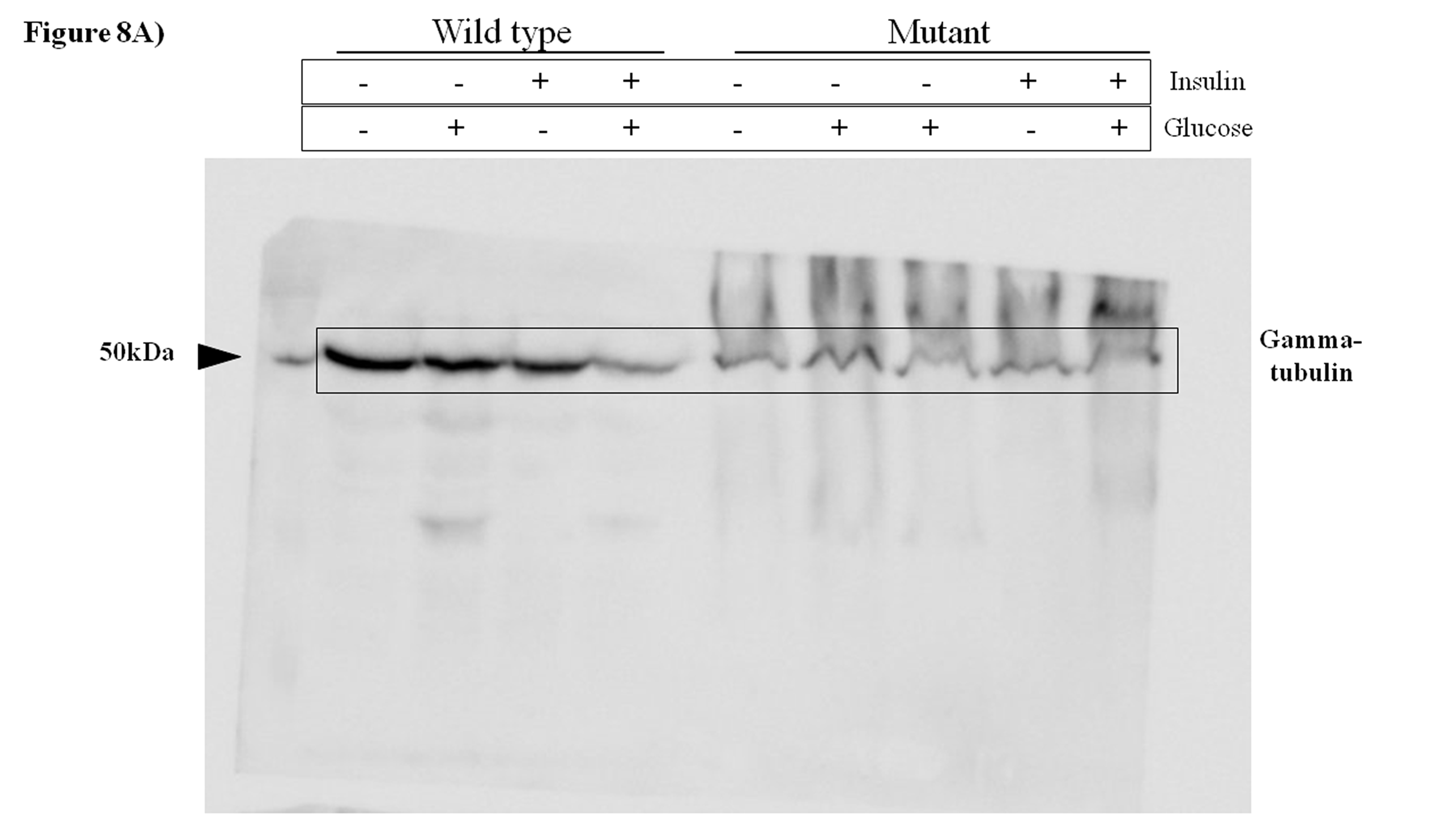

Supplement: Supplementary Figure 8A2 [file mmc3.jpg]

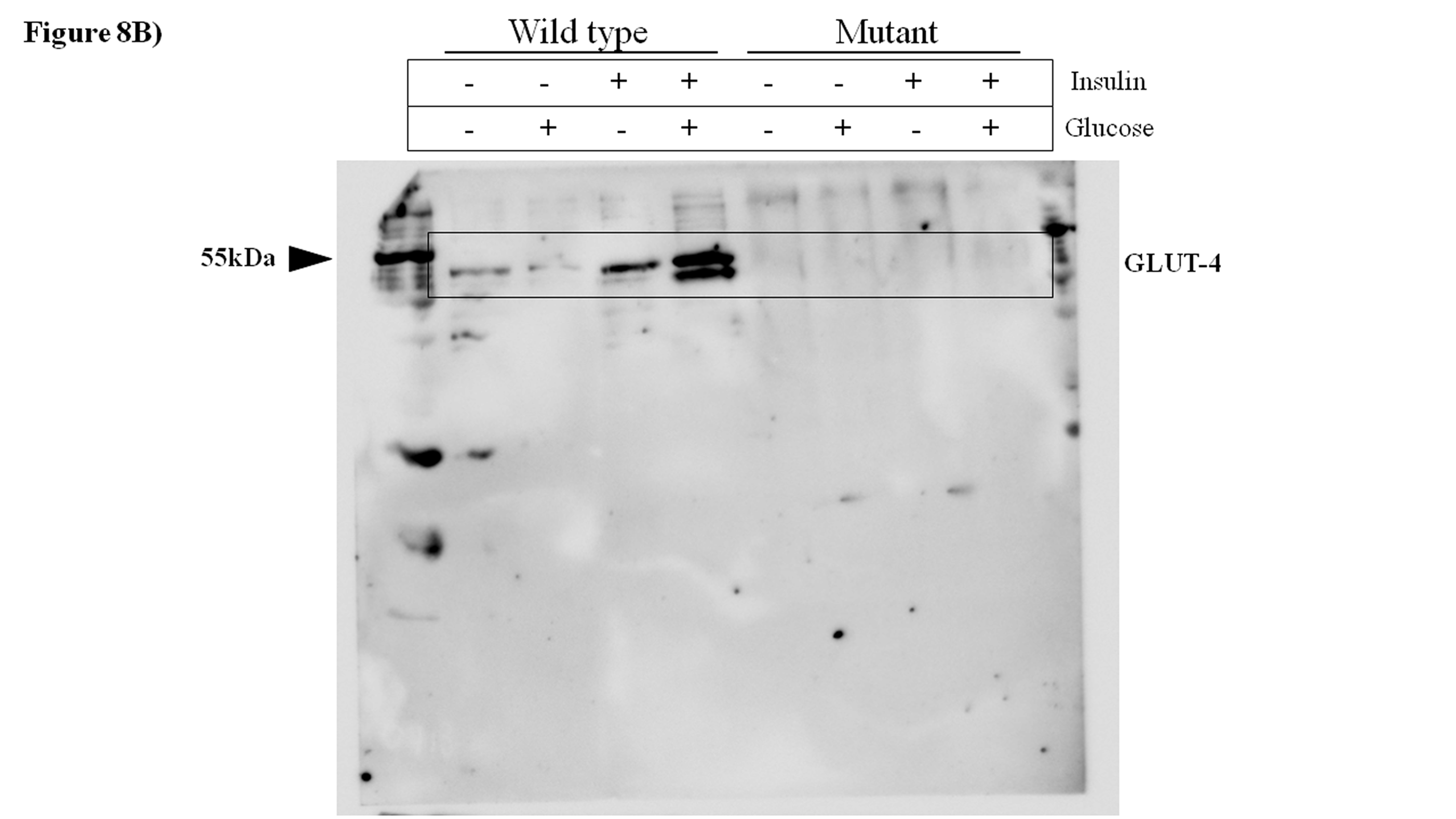

Supplement: Supplementary Figure 8B1 [file mmc4.jpg]

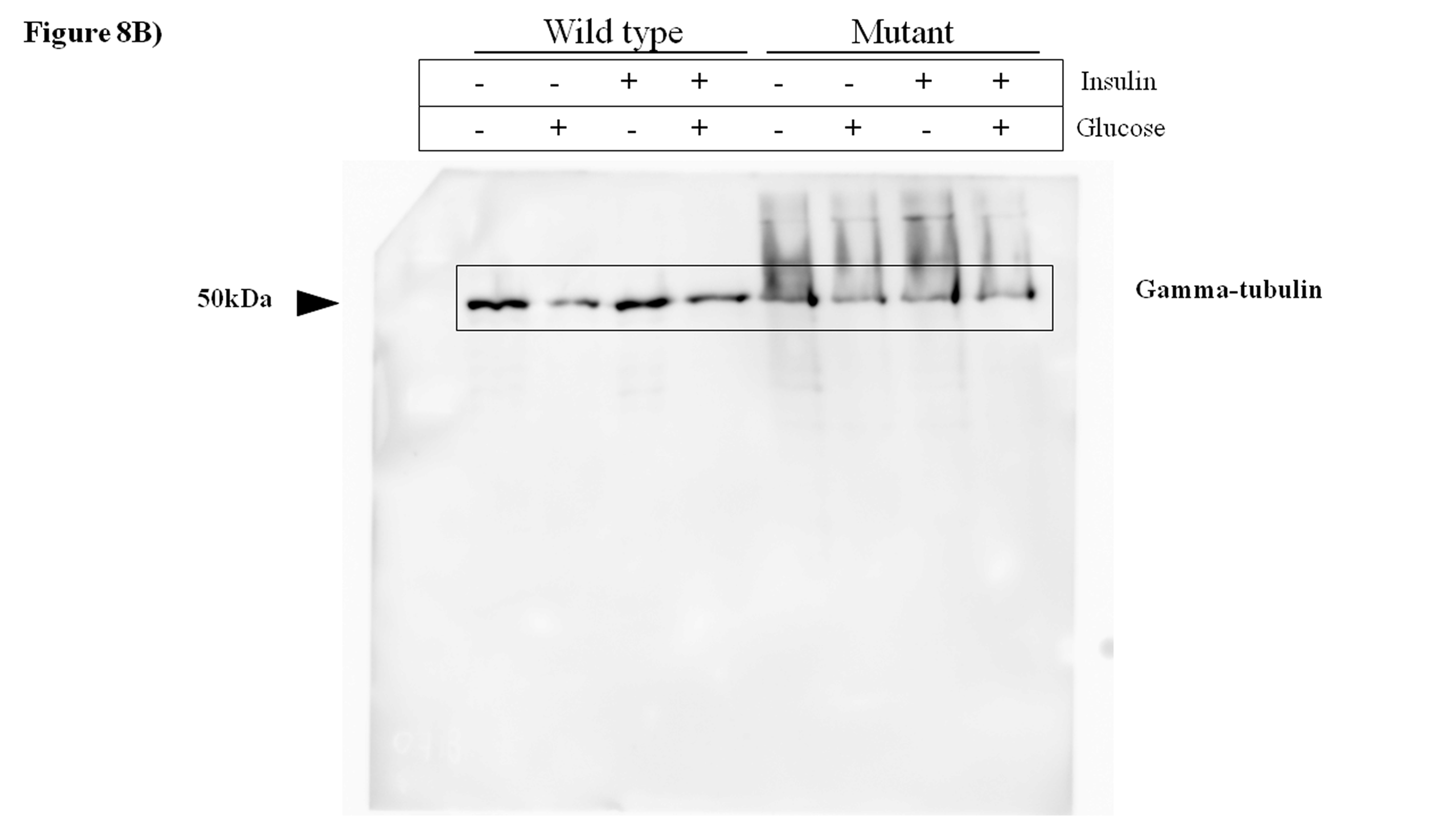

Supplement: Supplementary Figure 8B2 [file mmc5.jpg]

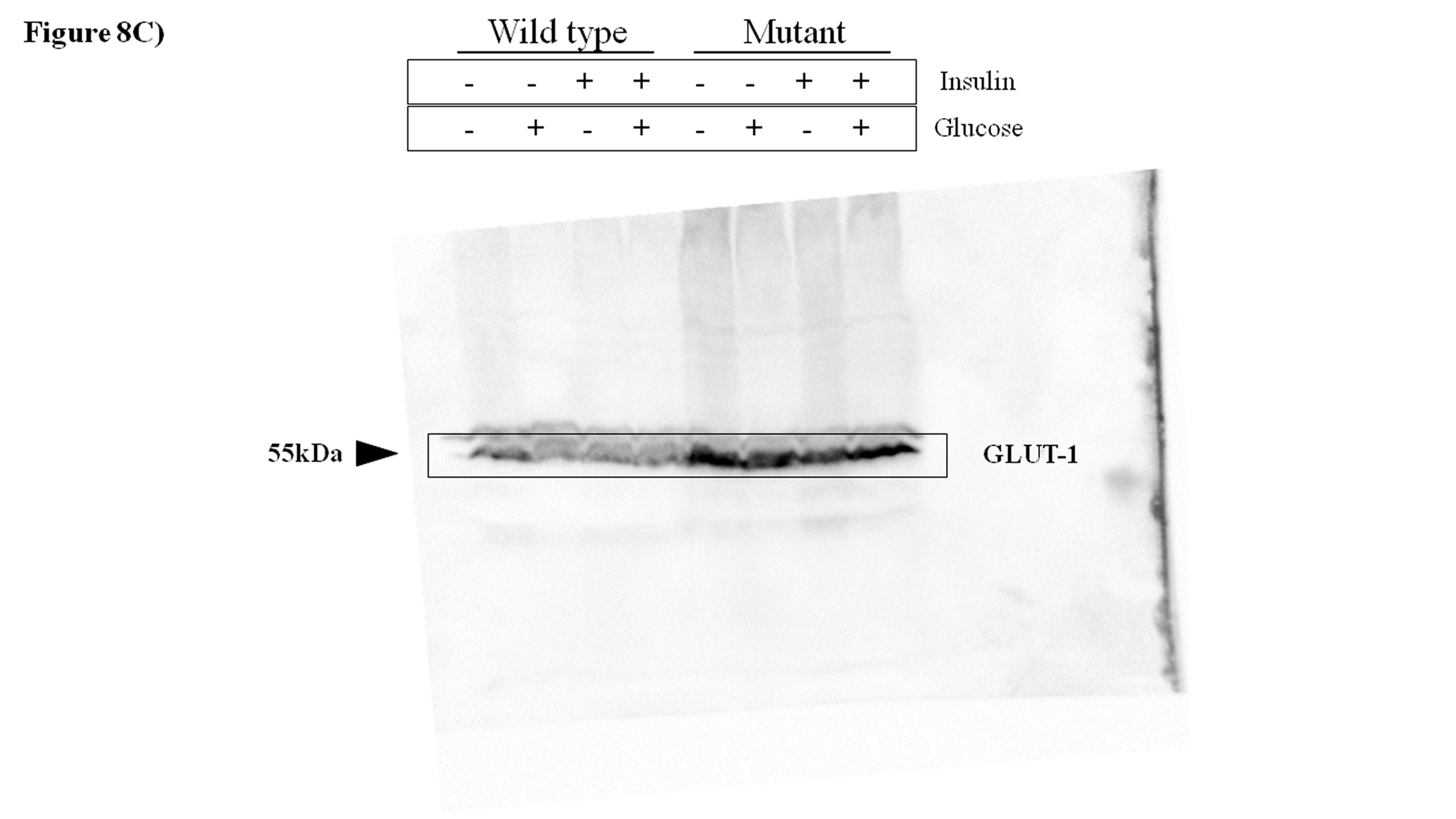

Supplement: Supplementary Figure 8C1 [file mmc6.jpg]

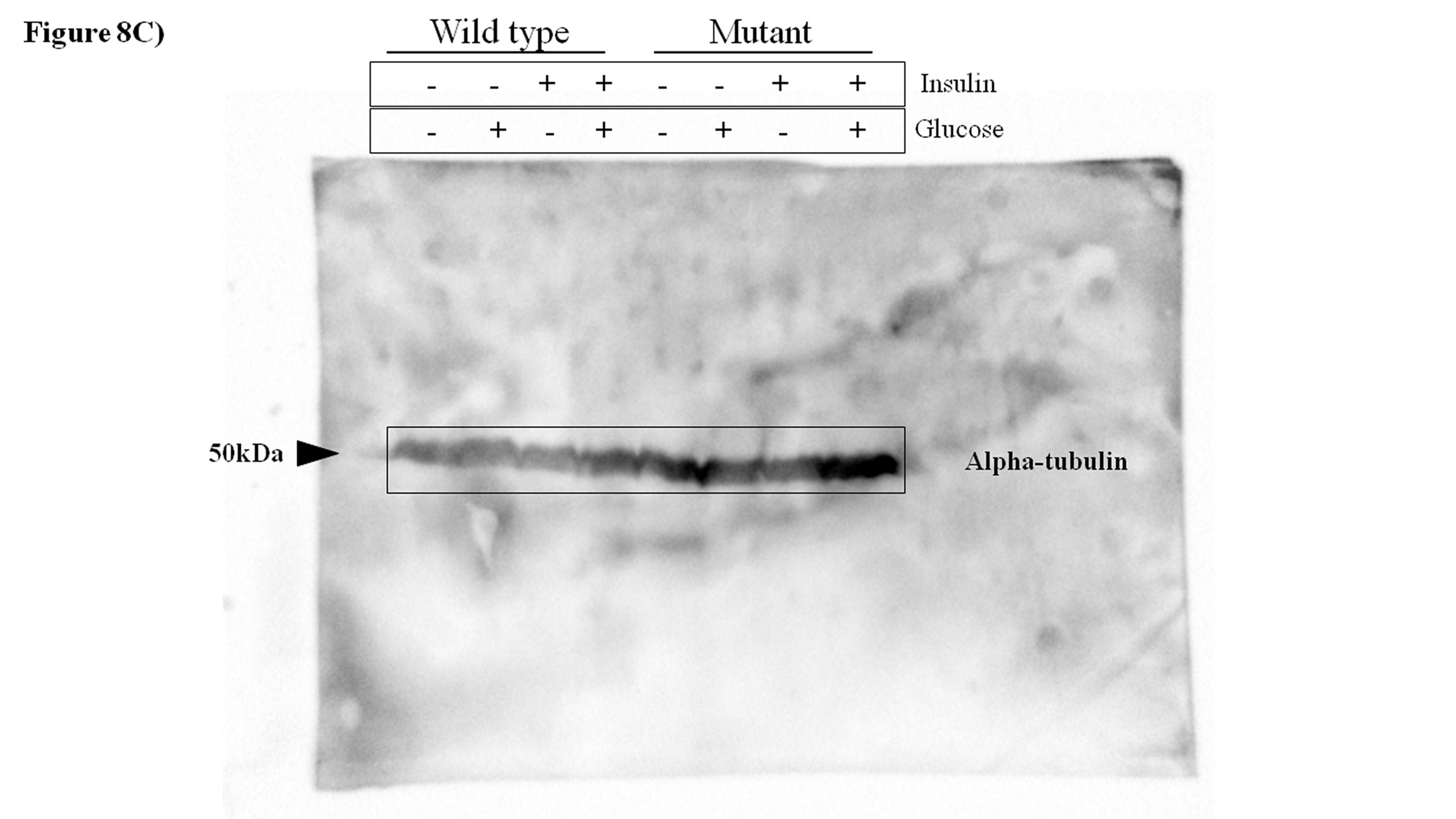

Supplement: Supplementary Figure 8C2 [file mmc7.jpg]

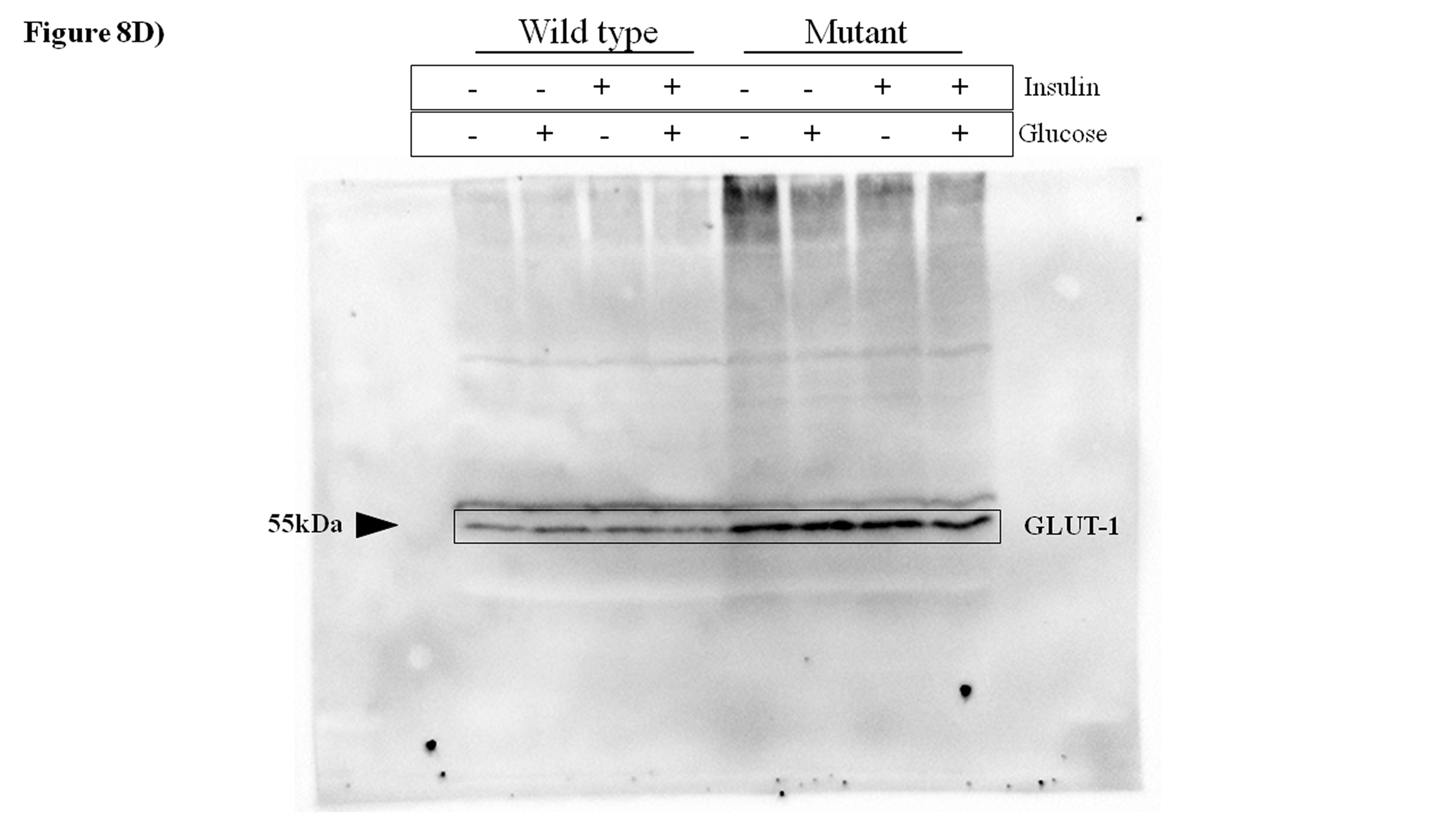

Supplement: Supplementary Figure 8D1 [file mmc8.jpg]

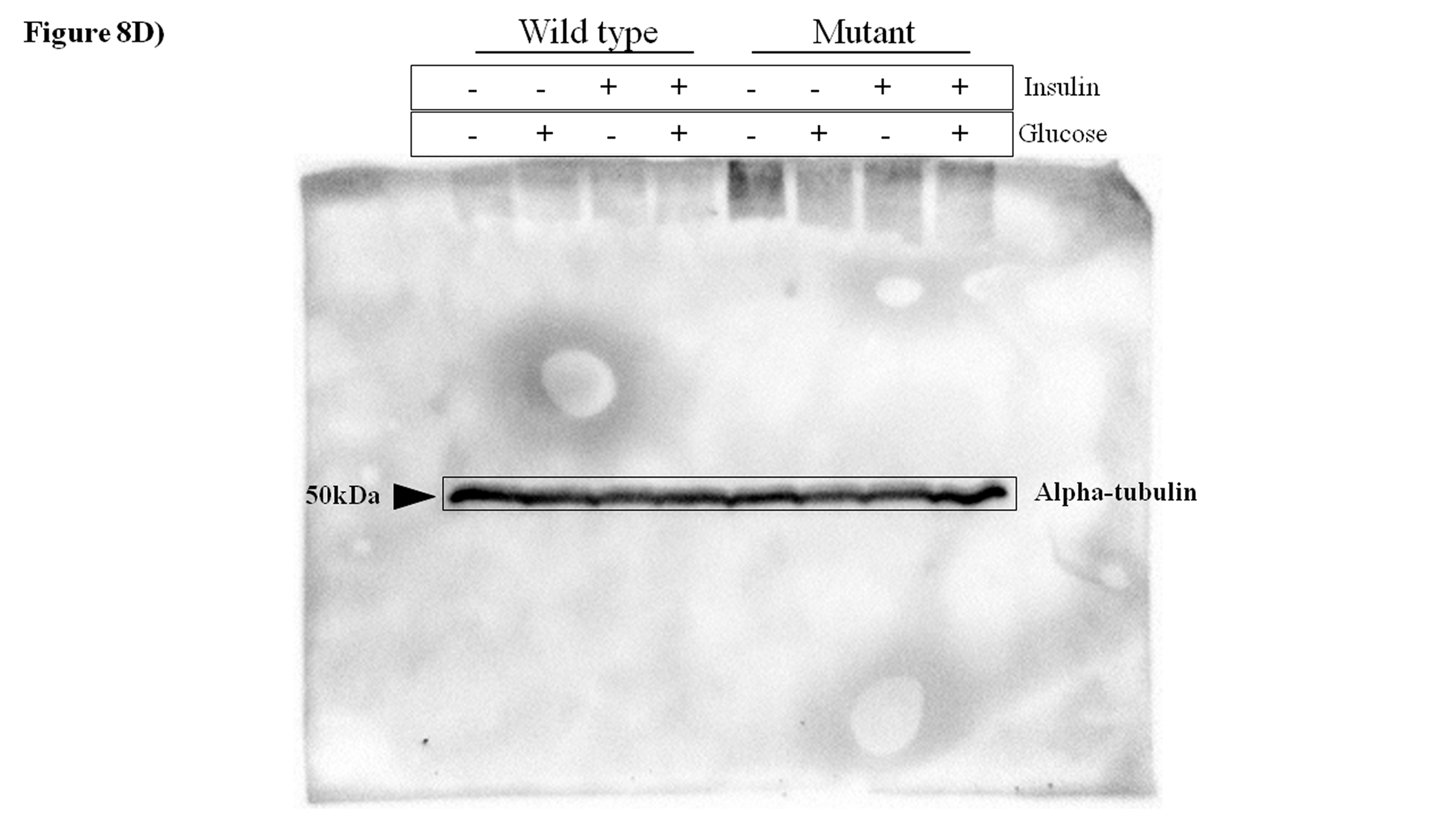

Supplement: Supplementary Figure 8D2 [file mmc9.jpg]
